# High-Resolution Melting of 12S rRNA and Cytochrome *b* DNA Sequences for Discrimination of Species within Distinct European Animal Families

**DOI:** 10.1371/journal.pone.0115575

**Published:** 2014-12-22

**Authors:** Jana Naue, Tobias Hansmann, Ulrike Schmidt

**Affiliations:** 1 Institute of Legal Medicine, Freiburg University Medical Center, Albertstrasse 9, D-79104 Freiburg, Germany; 2 Faculty of Biology, University of Freiburg, Schaenzlestrasse 1, D-79104 Freiburg, Germany; University of New England, Australia

## Abstract

The cheap and easy identification of species is necessary within multiple fields of molecular biology. The use of high-resolution melting (HRM) of DNA provides a fast closed-tube method for analysis of the sequence composition of the mitochondrial genes 12S rRNA and cytochrome *b*. We investigated the potential use of HRM for species identification within eleven different animal groups commonly found in Europe by animal-group-specific DNA amplification followed by DNA melting. Influence factors as DNA amount, additional single base alterations, and the existence of mixed samples were taken into consideration. Visual inspection combined with mathematical evaluation of the curve shapes did resolve nearly all species within an animal group. The assay can therefore not only be used for identification of animal groups and mixture analysis but also for species identification within the respective groups. The use of a universal 12S rRNA system additionally revealed a possible approach for species discrimination, mostly by exclusion. The use of the HRM assay showed to be a reliable, fast, and cheap method for species discrimination within a broad range of different animal species and can be used in a flexible “modular” manner depending on the question to be solved.

## Introduction

During the last years, analysis of non-human DNA got more important especially in the fields of food analysis, forensic science, and ecological studies [1]–[4]. Species identification is widely performed by using standard methods as Sanger sequencing and Restriction fragment length polymorphism (RFLP) methods [5] analyzing mostly mitochondrial (mt) genes for cytochrome *b* (cytb), 12S ribosomal ribonucleic acid (12S rRNA) or cytochrome oxidase I (COXI) [5]–[11]. Both methods are time-consuming and susceptible to contamination due to a multi-step process. Closed-tube assays as a Real-Time PCR followed by a melting curve analysis (MCA) avoid these drawbacks and are cheap alternatives. Applications are reported e. g. in the field of food chemistry but also in forensic biology [3], [12]. The melt curve analysis uses the possibility of differentiating PCR products by their characteristic melting temperatures (T_m_) at which the transition of dsDNA to ssDNA occurs depending on fragment length and sequence composition [13], [14]. Enhanced technologies as high-resolution melting (HRM) improve the temperature resolution down to 0.1°C/step and thereby lead to a more accurate monitoring of the melting behavior increasing the discrimination of sequence variants between PCR products. Additionally, improved analysis methods do not only consider the absolute T_m_ but also the shape of the melting curve, facilitating PCR fragment differentiation.

Recently, we published a nested Real-time PCR assay based on a universal pre-amplification of 12S rRNA and cytb PCR fragments. PCR products were then used for a parallel specific main-amplification of 10 distinct groups of animals (the common European animal families Phasianidae, Equidae, Cervidae, Canidae, Leporidae, Felidae, and Mustelidae as well as the tribe Bovini, and the two species *Homo sapiens* and *Sus scrofa domestica*) and were analyzed by MCA [12]. Due to the heterogeneous composition of target species (7 families, one tribe, and two species), the term “animal groups” will be used in this paper. Here we describe the possibilities of further discriminating species within the animal groups mentioned by exploiting inter-species differences of the T_m_. Additionally, The assay was extended for the sub-family Caprinae and modified for the use on a Rotor Gene Q allowing HRM analysis. In addition to visual and mathematical evaluation (by root-mean-square error (RMSE) analysis) of inter-species differences, reproducibility, and discrepancies due to intra-species differences were taken into consideration and mixture analysis was tested. With HRM, no further reaction or analysis steps are necessary achieving the same advantages as in MCA, but with a considerable gain in information on unknown samples. HRM analysis provides a simple, cheap, and quick tool for analysis of non-human DNA.

## Materials and Methods

All kits were used according to the manufacturers' recommendations unless otherwise stated.

### Samples, DNA extraction, quantification and sequence validation

Blood and muscle samples were collected from deceased animals that had been examined at the Department of Animal Hygiene, Chemical and Veterinary Investigatory Office in Freiburg. Human and donkey DNA was obtained from buccal swabs (Sarstedt, Hildesheim, Germany). DNA analysis was approved by the Ethics Committee of the University of Freiburg (Approval Number: 419/09_130559). DNA was extracted by Phenol-Chloroform or the Qiagen Mini Kit (Qiagen, Hilden, Germany), and was mostly eluted in water. Additionally, DNA extracts (1xTE elution buffer after PureGene extraction) of several animals were obtained from the Institute of Veterinary Pathology, University of Giessen. DNA of tissue samples was extracted and quantified as reported before [12]. DNA sources (S1 Table) were verified by sequencing 404–419-bp (12S rRNA) and 483–488-bp (cytb) fragments surrounding the target regions using universal primers as previously reported [12]. Due to initially unsuccessful amplification or to co-amplification of pseudogenes, some of the DNA samples were sequenced using the Pre-amplification primers of Naue et al. (2014) resulting in shorter fragments (around 200 bp for 12S rRNA and 224 bp for cytb). DNA sequences were verified by the NCBI database search for local alignments (blast.ncbi.nlm.nih.gov/Blast.cgi). An overview of all 118 samples included in the HRM analysis and the reference sequences used for alignment are provided in S1 and S2 Tables. Sequences of 12S rRNA and cytb for all samples with >200 bp were submitted to GenBank [http://www.ncbi.nlm.nih.gov/, accession numbers KM224229-KM224433]. Not all the known species contained in an animal group were considered for analysis due to limitations in sampling and to reliable and successful amplification (e. g. too many differences in the primer sequence led to the exclusion of water buffalo from the Bovini tribe assay).

### Real-Time PCR Assay

Real-time PCR and HRM were performed on a Rotor-Gene Q 5-Plex HRM (RGQ) with Rotor-Gene-Pure Detection software v2.2.3 (Qiagen).

If applicable, singleplex reactions for 12S rRNA and cytb were done by using 5 ng of DNA eluted in water. From samples eluted in TE, 1 µL of a 1∶20 dilution was inserted to maintain the ion concentration of the reaction buffer. The reaction mixes contained 1xHRM PCR Master Mix (Qiagen) and 0.2–0.4 µM of each primer (Biomers, Hilden; primers according to [8], [12]; Caprinae: 12S rRNA: forward: CGTAAAGCGTGTTAAAGCATCATACT and CGTAAAACGTGTTAAAGCACTACATC, reverse: TGGGTCTTAGCTATGGTGTATCAG; cytb: forward: TATATTGGCACAAACCTAGTCGAA, reverse: GAGGGCTGTGATGATGAATGGG) Water was added to a final volume of 20 µL. Cycling was 95°C for 5 min, 11 cycles with 95°C for 45 s and 66°C to 64°C (0.2°C/cycle) for 80 s, followed by 24 cycles with 95°C for 45 s and 64°C for 80 s. HRM from 65°C to 90°C with 0.1°C/step and a wait of 2 s after each step was performed after amplification. Fluorescence data were collected on the green channel during cycling and on the HRM channel during the melting step. Non-template controls (NTC) were carried along.

### Data interpretation

Data were analyzed by using the quantitation, melt, and HRM analysis tools of the RGQ software. Only samples successfully amplified with a Ct lower than 30 were considered for further analysis. The threshold for Ct calculation and the normalization regions (leading and trailing ranges) for HRM analysis were independently defined for 12S rRNA and cytb of each animal group as well as for the universal 12S rRNA assay.

### Curve interpretation and comparison

Curve shapes between samples of the same species, between species, and of samples with mutations were compared. Therefore, the normalized fluorescence data were used to calculate the root-mean square error (RMSE) values for each sample pair using Matlab (MathWorks, Ismaning, Germany) to assess the shape similarities or differences of the obtained melting curves. RMSE for intra-species sample similarity was not calculated for species only represented by one single sample (arctic fox, zebu, and reindeer), and for species with less than 2 successfully amplified samples (yak: 12S rRNA; ferret, beech marten, and badger: cytb).

### Reproducibility

For an evaluation of intra- and inter-run accuracy of the HRM results of both genes, three runs of triplicates and an iteration of Phasianidae, Equidae and Caprinae samples were performed, and the RMSE values calculated as described.

### Influence of template DNA

The influence of template DNA was exemplarily investigated for Phasianidae samples due to observed deviations during analysis. Depending on the DNA concentration obtained during DNA extraction, DNA amounts of 50 ng (Chicken) or 36 ng (Turkey), 10 ng, 5 ng, 1 ng, 250 pg, and100 pg were used in triplicates for HRM.

### Mixtures

As examples of inter-species/animal-group mixtures, 50-%, 20-%, and 10-% mixtures of human and pig were generated and were analyzed with universal 12S rRNA primers. Additionally, exemplary mixtures of species within the same animal group were prepared and analyzed with animal group specific primers. These were Chicken/Turkey (Phasianidae), Sheep/Goat (Caprinae), and Hare/Rabbit (Leporidae).

### Testing of blind samples

Ten unknown DNA samples - eight single-source samples (1 ng) and two two-source mixtures with a 50∶50 ratio (each 1 ng) - were analyzed by HRM.

## Results

To investigate the potential use of HRM for species identification, we analyzed animals covering species of eleven different animal (sub-)families, tribes or species abundant in Europe: Phasianidae, Equidae, Cervidae, Canidae, Leporidae, Felidae, Mustelidae, the tribe Bovini, the subfamily Caprinae, and the two species *Homo sapiens* and *Sus scrofa (domestica)*.

### Accuracy and reproducibility

The evaluation of intra- and inter-run accuracy for samples of the Phasianidae, Caprinae and Equidae revealed comparable and low RMSE (Table 1). The grand mean RMSE represents the average of the RMSE means of triplicates over the different samples. Additionally, the maximum observed RMSE of one sample within one run and over three runs is given. A mean deviation lower than 1% of the normalized fluorescence values was found between triplicates within the same run, with a maximum observed RMSE of 1.72 when analyzing the cytochrome *b* gene of the Phasianidae. The results show a slightly higher deviation between the melting curves of a given sample in different runs than within the same run.

**Table 1:** Reproducibility of HRM curves within and between three runs.

**Phasianidae (9 samples)**	Gene	Grand mean RMSE	Max RMSE
intra-run variation	12S rRNA	0.46	0.84
	CYTB	0.7	1.72
inter-run variation	12S rRNA	0.84	1.52
	CYTB	0.97	1.76
samples: 5 chicken, 4 turkeys			
**Equidae (5 samples)**	Gene	Grand mean RMSE	Max RMSE
intra-run variation	12S rRNA	0.75	1.34
	CYTB	0.36	0.84
inter-run variation	12S rRNA	1.08	1.84
	CYTB	0.71	1.64
samples: 3 horses, 2 donkey			
**Caprinae (6 samples)**	Gene	Grand mean RMSE	Max RMSE
intra-run variation	12S rRNA	0.55	1.8
	CYTB	0.4	0.93
inter-run variation	12S rRNA	0.83	2.67
	CYTB	0.41	0.90
samples: 3 goats, 3 sheeps			

The RMSE values between all measured samples were calculated and the mean values of all triplicate means (grand mean RMSE), as well as the maximal observed RMSE are presented.

Additionally, we calculated RMSE values for all samples from each species included in the 11 animal groups (cf. S1 Fig.). Regarding all observed RMSE values between samples of the same species, the values for 12S rRNA were within a range from 0.058 to 2.9 with the exception of cats (RMSE value of up to 7.4). For cytb the values ranged between 0.08–2.976 with the outliers lynx (RMSE up to 6.1), roe deer (RMSE up to 11.2), and fallow deer (RMSE up to 4.2). Due to the limited and differing sample sizes of each species analyzed, these results are exemplary.

### Inter-species discrimination

We used at least three samples of the same species with the same DNA sequence for comparison whenever available. Furthermore, we tested samples with individual single base mutations to observe their influence on the melting curve and potential difficulties in species classification. All information on the samples and results including sequence mutations and normalized HRM graphs can be found in S1 Table and S1 Fig. Furthermore, the reference sequences of all species within each animal group analyzed were compared as to their nucleotide differences within the species specific 12S rRNA und cytb fragments as well as the universal 12S rRNA fragment. Thereby, information about the roughly expected melting behavior due to the fragments' GC content and the observed melting behavior were compared and revealed good concordance (S2 Table).

**Figure 1:**
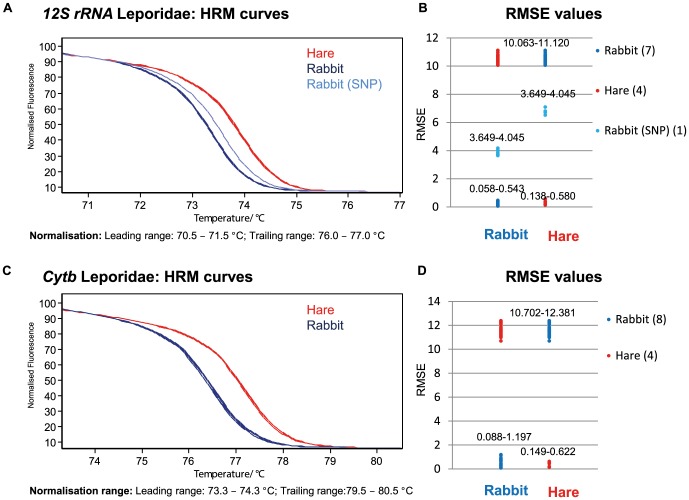
Melt curves and RMSE values for animal-family specific PCR and HRM of the Leporidae. A) Normalized HRM results for 12S rRNA. The SNP represents an A>G transition. B) RMSE value range for the comparison of all curves presented in A. C) Normalized HRM results for cytb. D) RMSE value range for the comparison of all curves presented in C. Lower values were calculated for samples of the same species and species differentiation is possible. The SNP does not fit into the range obtained for the 12S rRNA rabbit fragments, but does not interfere with the hare curves.

**Figure 2:**
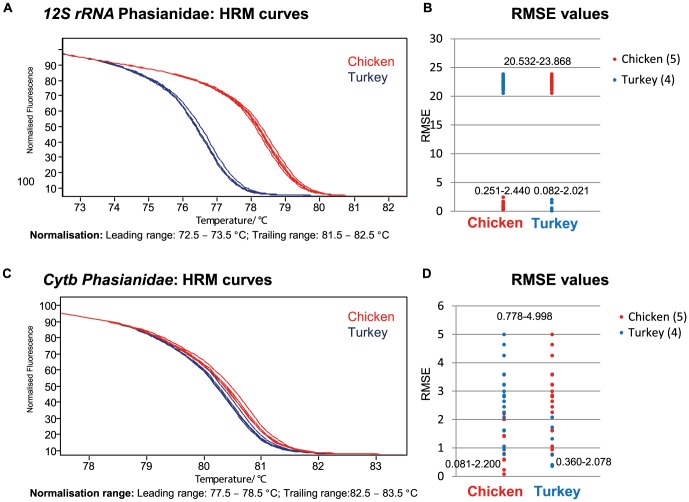
Melt curves and RMSE values for animal-family specific PCR and HRM of the Phasianidae. A) Normalized HRM results for 12S rRNA. B) RMSE value range for the comparison of all curves presented in A. C) Normalized HRM results for cytb. D) RMSE value range for the comparison of all curves presented in C. Lower values were calculated for samples of the same species within 12S rRNA and species differentiation is possible. Analyzing cytb only would lead to more inconclusive results, although a slightly different melting behavior is also visible.

Two approaches were used to explore the species discrimination within each animal group: (1) visual inspection and (2) differentiation using the calculated RMSE values (cf. S1 Fig.). Figs. 1 (Leporidae) and 2 (Phasianidae) give examples for clear and inconclusive species classification depicting melt curves (Fig. 1A,C and 2 A,C) and the obtained RMSE values (Fig. 1B,D and 2B,D). Within the Leporidae, a visual and mathematical distinction between hare and rabbit is easily possible for 12S rRNA and cytb. As expected, an A>G mutation within one rabbit sample leads to an increased melting temperature due to the higher GC content without interfering with the hare samples' melting curves (Fig. 1A,C). A similar alteration can be observed for a hare sample (T>C) analyzed with the universal 12S rRNA primers (cf. S2H Fig.). A discrimination of chicken and turkey is only reliably possible by 12S rRNA analysis (Fig. 2A and B) but not by cytb (Fig. 2C and D), although the chicken samples tend to have higher T_m_.

**Figure 3:**
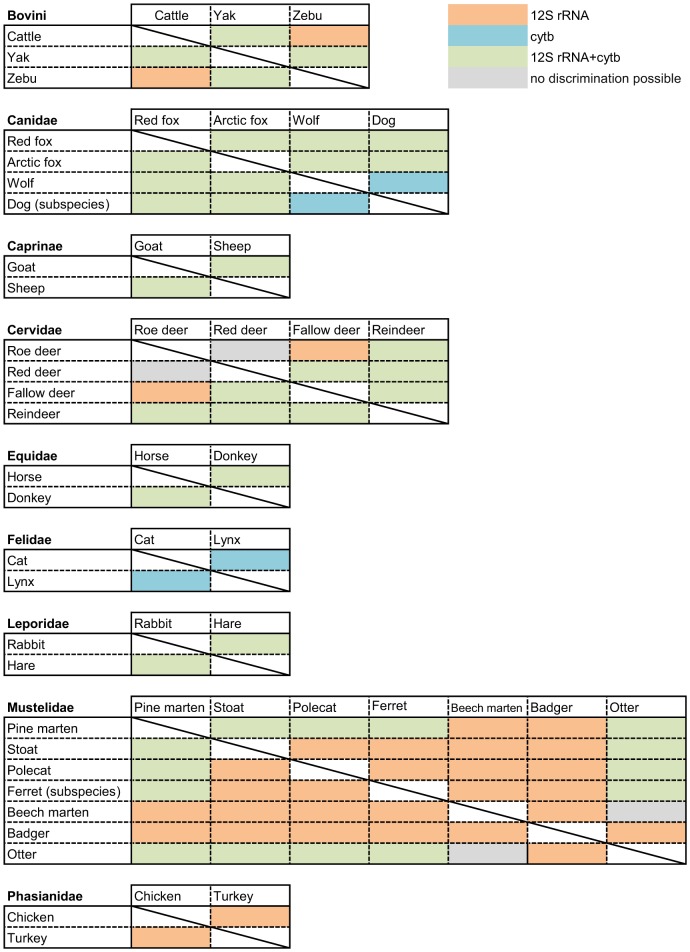
Summary of possible visual discriminations within the animal groups analyzed. Except for the pairs roe deer/red deer and beech marten/otter, discrimination was always possible by the analysis of at least one gene. Within the Mustelidae, the discrimination relies mostly on 12S rRNA, as no successful amplification for cytb was possible for all species.

We were able to differentiate almost all species (exceptions: Red deer/roe deer and otter/beech marten; cf. S1D and I Fig., Fig. 3) by at least one of the two gene fragments investigated, and outliers are mostly explained by sequence mutations or variations in DNA template amounts. As expected due to the same nucleotide sequence, no differentiation was possible for cytb from cattle and zebu. The RMSE values objectively confirmed the visual difference of melt curves as well as the lacking discrimination of some samples; e. g. cytb analysis of chicken and turkey (Fig. 2C, D). Differentiation was possible by either both 12S rRNA and cytb or by analysis of either 12S rRNA or cytb. A summary is given in Fig. 3. In some cases, a sequence mutation shifted the melting profile to a degree that the resulting curve fitted none of the species profiles (e. g. one horse sample in cytb (Equidae), two pine marten and one stoat samples in 12S rRNA (Mustelidae; cf. S1E and I Fig.). However, a sequence mutation never made a melting curve fitting the curves of a different species included in the assay (cf. intra-species variation).

### Intra-species variation

Single nucleotide polymorphisms (SNPs) can occur within species and have to be considered as a factor affecting the melting behavior. We saw no effect in case of an A insertion within pig samples (cf. S1K Fig.). On the other hand, transitions did lead to a shift of the melting curve depending on the mutation: A>G increased the melting temperature, whereas C>T resulted in a decrease. Interestingly, an A>G mutation within the cytb gene enabled successful discrimination of dog and wolf (S1B Fig.). Although a sequence mutation within the primer region does not change the resulting sequence composition of the PCR fragment, a decrease of the amplification efficiency was observed (but still Ct<30) for one stoat sample of the Mustelidae family resulting in a shift of the melting curve (cf. S1I Fig., 12S rRNA).

### Discrimination by universal 12S rRNA primer

**Figure 4:**
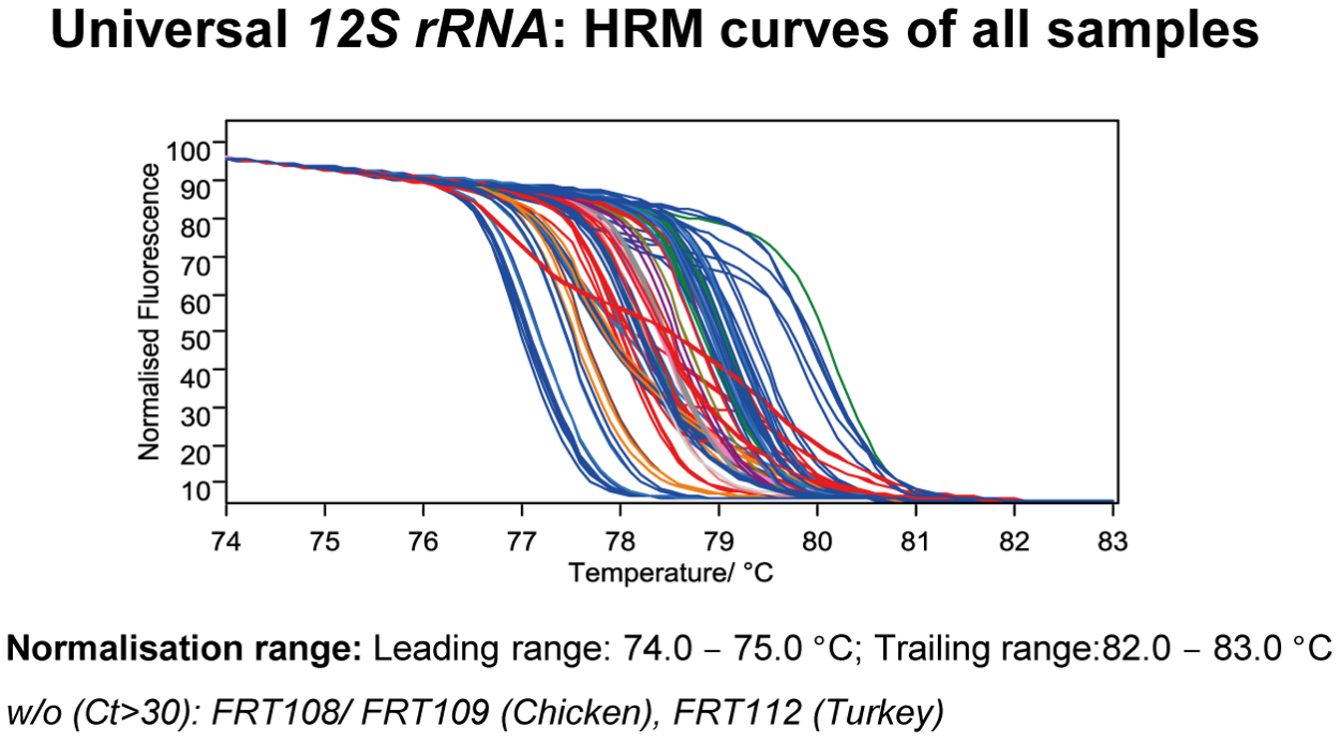
Normalized melt curves using the universal 12S rRNA primers. The curves of two runs (necessary due to sample number) were merged. A distribution of the T_m_s over 3°C can be observed as well as special melt curve features resulting in direct discrimination, e.g. sheep. Compare to S2 Fig.

We also tested if universal 12S rRNA primers [8] are sufficient for species discrimination by HRM. The T_m_ of all analyzed samples varied within a temperature range from 77 to 80°C (Fig. 4) and most species were readily identified within their animal group (S2 Fig.). Some species are directly discriminable without any further knowledge on the animal group due to their particular melting profiles (more complex than the expected melting behavior (S2 Table)), probably caused by a co-amplification of nuclear mitochondrial DNA (NUMTS) (e. g. sheep – cf. S2C Fig.). Vice versa, differences or special features of the melt curve can directly be used for exclusion of some species due to melt curve discrepancies. A disagreement to the observed melt curves was only obtained for chicken/turkey discrimination.

### Effect of elution buffer/DNA concentration

**Figure 5:**
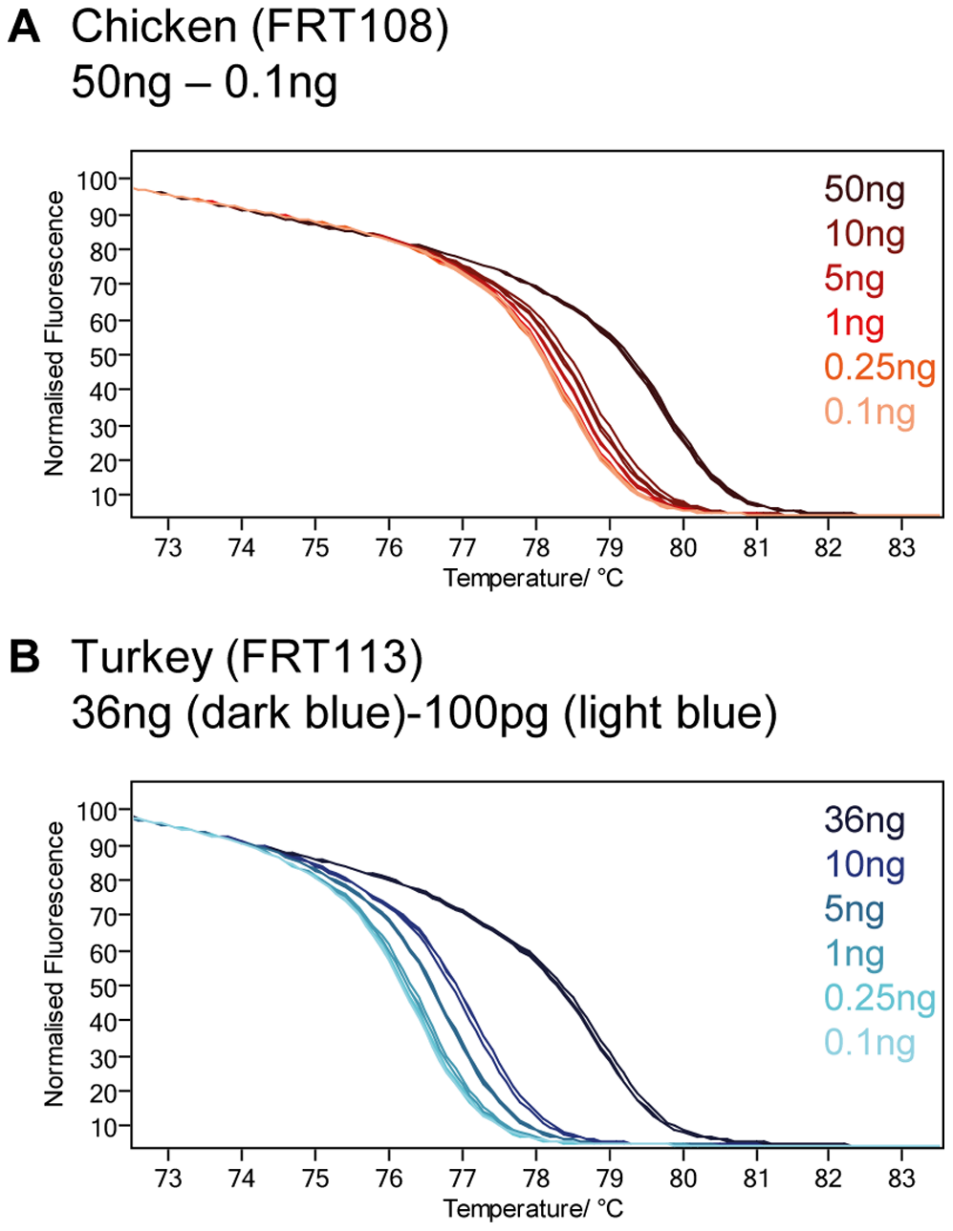
Impact of DNA amount used for PCR. Two samples were used for the PCR reaction in different dilution steps. A) A dilution from 50 ng down to 100 pg of a chicken DNA sample was analyzed. The highest deviation is visible for 50 ng. B) A dilution from 36 ng down to 100 pg of a turkey DNA sample was analyzed. The highest deviation is visible for 36 ng followed by 10 ng and 5 ng.

DNA samples eluted in a TE buffer resulted in a T_m_ shift compared to samples eluted in water hampering interpretation of results. Therefore, DNA samples eluted in either water or in TE, were analyzed together within one animal group. Generally, DNA amount was adjusted to 5 ng, except for samples eluted in TE; here, the total amount of buffer was found to be more interfering than differing DNA amounts. In some samples, this was not possible due to a very low DNA content. We tested the effect of varying DNA amounts with a chicken and a turkey sample (Fig. 5). A considerable shift in T_m_ and the melt curve was especially observed for high DNA amounts of 36 ng (mean RMSE of triplicates 16.8846–24.0218) and 50 ng (RMSE 10.546–13.7971). DNA amounts between 100 pg and 5 ng resulted in a lower deviation for chicken (RMSE 1.7848–0.0788) and higher values for turkey (RMSE 5.4315-0.272). Using a maximum of 5 ng of template DNA, we lowered the impact of varying DNA concentrations on HRM assay, but since only quantification of total DNA was performed, discrepancies due to different amounts of mtDNA can still alter the melting behavior.

### Mixtures

**Figure 6:**
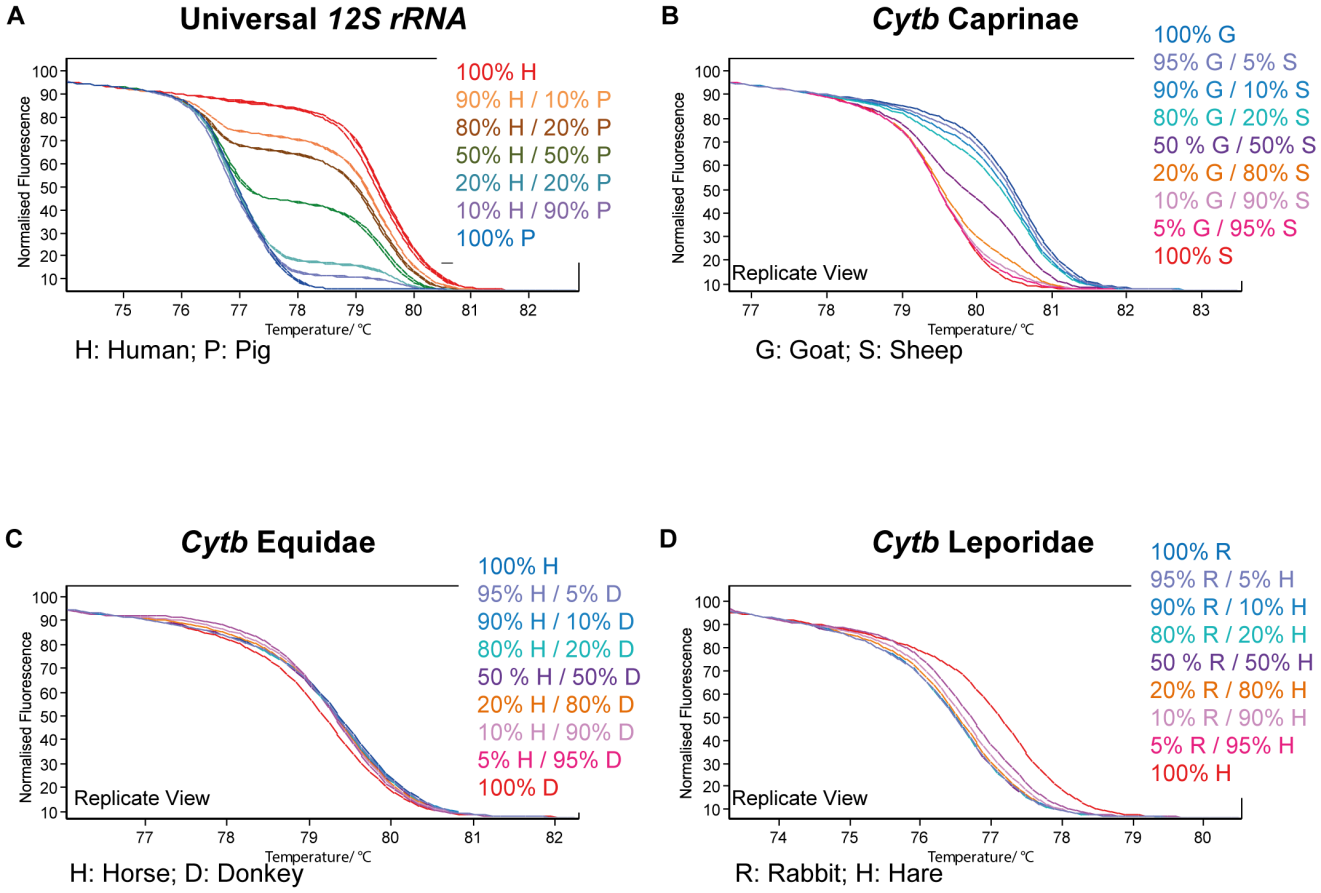
Mixture analysis within and between animal groups. A) Mixtures of pig and human DNA representing animals of two different groups can be successfully resolved. B–D) Mixtures between samples of the same animal group i.e. family. The success of mixture resolution varied between the animal groups.

Mixtures between the two different species human and pig were measured by using the universal 12S rRNA primers and revealed results comparable to the discrimination power obtained with the Real-Time PCR assay ([12]; cf. Fig. 6A).

In case of sheep/goat, analysis of cytb allows for species discrimination (Fig. 6B). Mixtures of species belonging to the same animal group were dissolved either by two-domain melting or a less sharp transition rate, but did show limitations: (1) although the same genomic DNA amount was used, the donkey and horse mixtures show only melt curves consistent with horse. Due to sampling, horse DNA was obtained from muscle and donkey from buccal cells which results in a preponderance of horse mtDNA (Fig. 6C). (2) Target DNA from species of the same animal group compete for the primers, and may at the same time show different amplification efficiencies, e. g. 12S rRNA amplification of sheep and goat, or cytb amplification of hare and rabbit (Fig. 6D). All HRM curves of the mixtures tested for 12S rRNA, cytb and universal 12S rRNA are given in S3 Fig.

### Blind test

All of the ten single-source samples were correctly identified visually and mathematically by RMSE analysis as cow, goat, pig, fox, roe deer, turkey, cat, and horse. Also, the two-source samples were correctly identified as DNA mixtures from rabbit and badger, and from dog and beech marten (successful analysis of 12S rRNA only due to primer mismatches within cytb, cf. S1I Fig.). Initially, results for the dog and horse samples were visually inconclusive, since two samples with a SNP had been chosen by chance. Melt curves did not progress as anticipated and the calculated RMSE considerably deviated from the expected RMSE range in these two species. However, results still were clearly distinct from other species within the same group. RMSE and visual features also were identical to reference samples with known SNPs. Knowing the impact of the SNPs in the reference samples, both of the blind samples were positively assigned as mutated samples from a dog and a horse.

## Discussion

The use of Real-Time PCR for detection of various DNA samples is widely spread, e. g. diagnosis of genetic disorders or food analysis [3], [15]. Instead of using expensive analysis based on specific fluorescent probes, immediate post-PCR analysis by HRM provides a cheap alternative [4], [13], [16]–[18]. Next-generation or massively parallel sequencing is now more and more propagated as the favorable tool for any sequence analysis due to its high informational content. Nevertheless, especially screening tools need to be fast, reasonably cheap, and easy to handle. Particularly data interpretation has to be quick and reliable. Therefore, applications relying on alternative techniques that requiring a minimum of processing steps and costs but still providing the information needed are essential (e.g. compared in [19]). In this study, HRM analysis of eleven different animal groups (families, tribes, and species) was not only applicable for group identification but also for species discrimination. The combined analysis of two gene fragments led to a successful identification of all samples, except for roe deer/red deer and beech marten/otter, although not all the species were identified by both fragments (e. g. chicken/turkey; fallow deer/roe deer) as expected by sequence comparison (cf. S2 Table). Better discrimination can be expected for A:T to G:C changes as for the neutral changes A:T to T:A/G:C to C:G due to a change from two to three hydrogen bonds, leading to differences in T_m_ between 0.8°C and 1.4°C in small amplicons [20]. The effect of these changes can be erased by additional opposite mutations [3]. The expected melting behaviors (cf. S2 Table) were seen by HRM analysis. However, in case of universal 12S rRNA analysis, complex profiles for fox and sheep were obtained. They are explainable by co-amplification of NUMTs leading to a second melting domain. The only disagreement between expected and obtained melting behavior was seen for the universal 12S rRNA analysis of chicken and turkey. A higher T_m_ was expected for chicken than for turkey due to the higher amount of GC, but the impact of two insertions and one deletion may alter the expected T_m_ due to change of the fragment length and GC content. Additionally, amplification efficiency of these two species was impaired due to a higher amount of primer mismatches compared to all other species tested (cf. S1 Table) leading to HRM curves with higher intra-species deviations (cf. S2J Fig.).

Several compromises had to be made regarding the primer design that was aimed at (1) covering the highest possible number of different species within one animal group as well as (2) amplification of all species (across all animal groups) under the same PCR conditions. As a consequence, there was a weaker signal and a less reliable melt curve with higher intra-species deviations for the Felidae and for the cytb analysis of roe deer due to lower amplification efficiency. Also, there was successful cytb amplification in only five of the seven tested species of the Mustelidae (cf. Fig. 3, S1F and I Fig.). Species within the latter family are known for their high variability regarding cytochrome *b* [21]. The use of a primer mix to overcome problems of sequence variability in the primer binding regions (e. g. Canidae) also led to a change in the PCR fragment sequences between dog and fox (cf. S2 Table) additionally altering the melt curve which facilitated discrimination (cf. S1B Fig.). Additional single base mutations caused by transitions have to be considered carefully. In this study we observed mutations altering the melt curve shape, e. g. in samples from dog (cf. S1B Fig.12S rRNA and cytb), horse (cf. Dig. S1E cytb), and rabbit (cf. S1H Fig., 12S rRNA). Alterations of the melt curve reveal additional sequence mutations which however cannot be disclosed. This may lead to a (false) exclusion of a suspected species. In the presented setting, another (wrong) species call was not given. Nevertheless, it cannot be excluded that other sequence alterations not observed in this study or species not covered by our sample collection would generate overlapping melt curves and thus result in inaccurate sample assignment. The potential occurrence of additional mutations is already minimized by amplifying the small amplicons needed for good resolution in HRM [15], [22] as well as for successful analysis of degraded samples.

The method showed good inter-run stability, but stable buffer conditions and comparable amounts of template DNA should be used to obtain reliable results. The influence caused by the DNA amount, purity, and elution buffer is known but their impact seems to vary [13], [23], [24], as successful HRM analysis is also possible from non-quantified DNA obtained of dried blood samples [25] or from DNA concentrations differing by 3 orders of magnitude, as long as the plateau phase of PCR is reached [22]. The considerable differences between DNA amounts of 100 pg and 10 ng observed in our study can thereby be clarified, as the plateau phase was not reached in case of 100 pg and 250 pg DNA input. Nevertheless, the differences observed between the high DNA amounts (1 ng–36/50 ng) are not fully explained.

Resolution of DNA mixtures from individuals of the same animal group is especially effective when there are no primer-binding site mutations between the species included, since differing PCR efficiencies may lead to a total miss of one of the mixture components. Such a miss of a mixture component has to be considered in particular when samples are of different tissue types even when there are equal amounts of genomic DNA (cf. the horse/donkey mixture presented; cf. Fig. 6C). Depending on the sequence differences between the mixed species, two melting domains were sometimes observed (e. g. cytb Caprinae with 7 differences between goat and sheep, cf. Fig. 6B). If only a few nucleotides are different (e. g. cytb Equidae with 2 differences between horse and donkey), heteroduplex dsDNA structures between the mixed samples can occur during melting comparable to those obtained during analysis of nuclear heterozygous samples, leading to a melt profile with a broader transition region than the sharp transition in case of homozygous samples [13] or the non-mixed samples in our study (cf. Fig. 6C).

Data interpretation can be done visually, comparing the obtained normalized HRM melt curves. Additionally, the RMSE values used for interpretation of intra- and inter-run variability can be an alternative using their deviation range in each species as boundaries for classification which provides more objective criteria. In this study, the RMSE boundaries obtained mostly rely on a limited number of species samples, so they cannot be generally applied. For any laboratory-specific assay validation, a larger set of samples from a given species as well as the integration of a confidence interval for the boundaries determined should be used. Additionally, amplification products which show non-fitting HRM curves should always be sequenced to identify another species not covered by the controls and to differentiate the obtained result from a possible heteroduplex formation by a mixture. Single outliers with a RMSE near the endpoints of a given deviation range should be regarded carefully, as unexpected single base mutations, differing buffer conditions, and variable DNA amounts can also change the shape of the melt curve. Visual inspection and RMSE calculation should be used in combination, as the RMSE value does not provide information on the direction of a deviation to the reference melt curve. Use of the “light” digital filter implemented as standard setting in the operating software led to a slight smoothing of data eliminating technically caused fluorescence spikes. Comparing multiple runs, the same conditions of the analysis software have to be applied.

HRM analysis can be used as stand-alone screening tool, but reference controls should be taken along the process facilitating data interpretation. Having already a species “in mind” only the appropriate animal group has to be analyzed. Some species as the sheep can already be identified by universal 12S rRNA primers [8]. The universal 12S rRNA analysis can also provide an initial overview and constrict the potential species, thereby limiting further investigation to a minimum. On the other hand, analysis may directly end if a species under question can be excluded by the T_m_. To lower the costs for analysis of two genes, a combination to a multiplex assay might be helpful. Exemplarily, duplex reactions were tested for the family of Phasianidae. Successful differentiation was mostly possible by the 12S rRNA fragments' melting behavior as seen in the singleplex reactions (data not shown). Altogether, modular HRM analysis of 12S rRNA and cytb using family (group-) specific primers proved to be a simple and reliable screening tool for a broad range of European mammals as well as Phasianidae. This includes successful differentiation of 24 out of 28 species tested as well as the differentiation between canis lupus (wolf) and mustela putorius (polecat), and their respective subspecies canis lupus familiaris (dog) and mustela putorius furo (ferret).

## Supporting Information

S1 Fig **Normalized HRM curves and RMSE value ranges of animal-group-specific 12S rRNA and cytb analysis.**
(PDF)

S2 Fig **HRM analysis with the universal 12S rRNA primer.** The normalized HRM curves of two runs were merged into one figure. Additionally the curves were separated according to the animal groups.
(PDF)

S3 Fig **Normalized HRM curves for the group-specific12S rRNA, cytb and the universal 12S rRNA assays: analysis of different DNA mixtures.** Normalization ranges correspond to the ranges used for each animal group.
(PDF)

S1 Table **Overview of the samples used for analysis.** Species name, group assignment, accession numbers for the sequences, DNA amount, reference sequences for comparison and the differences to these are provided.
(PDF)

S2 Table **Comparison of base composition of PCR fragments within each animal group using the reference sequences.** Sequence differences are provided by annotation of the position and nucleotide within the reference sequence of species 1 in relation to position and nucleotide of species 2. Due to the change in GC content, causing different amounts of double and triple bonds, the hypothetical difference in T_m_ is given and compared to the observed T_m_.
(PDF)
