# Supplementary material for: High-Resolution Melting of 12S rRNA and Cytochrome b DNA Sequences for Discrimination of Species within Distinct European Animal Families
Source: PLoS One. 2014 Dec 22;9(12):e115575. doi: 10.1371/journal.pone.0115575 (PMC4274031; doi:10.1371/journal.pone.0115575)

Figure S1

A Bovini

12S rRNA: HRM curves

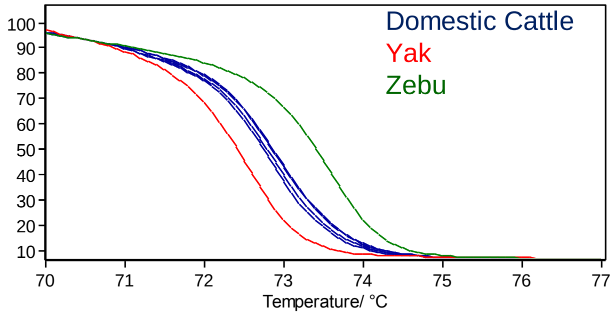

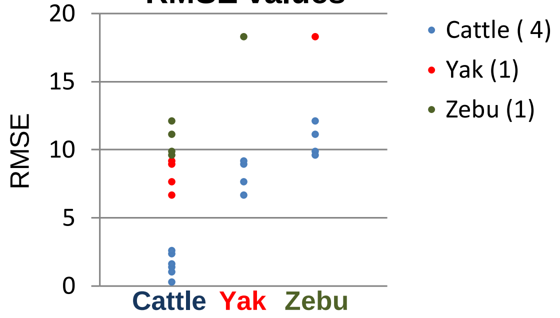

Normalisation: Leading range: 70.0 – 71.0 °C;
Trailing range: 76.0 – 77.0 °C

w/o (Ct>30): FRT6 (Yak)

Cytb: HRM curves

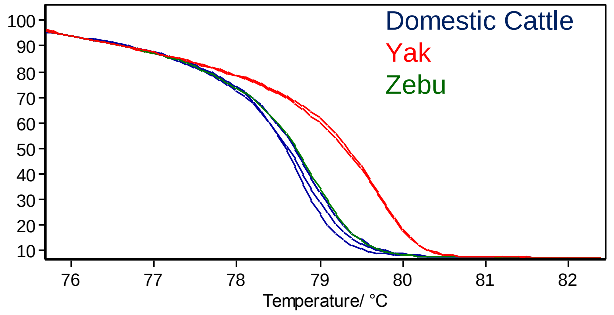

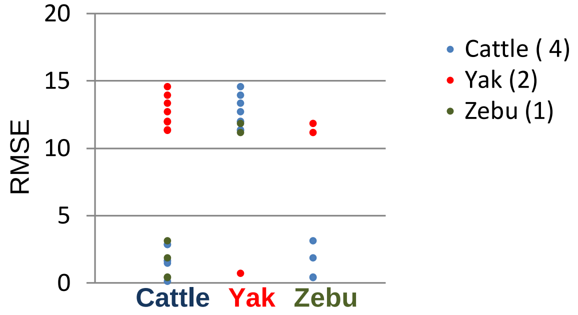

Normalisation: Leading range: 75.7 – 76.7 °C;
Trailing range: 81.4 – 82.4 °C

B Canidae

12S rRNA: HRM curves

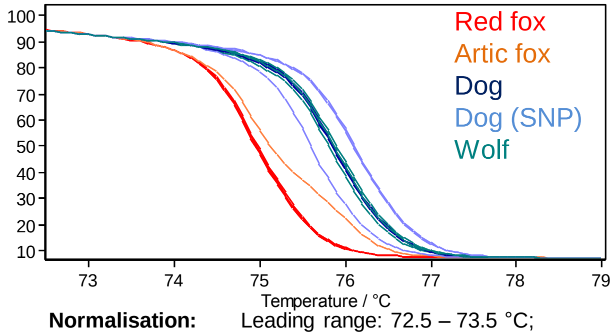

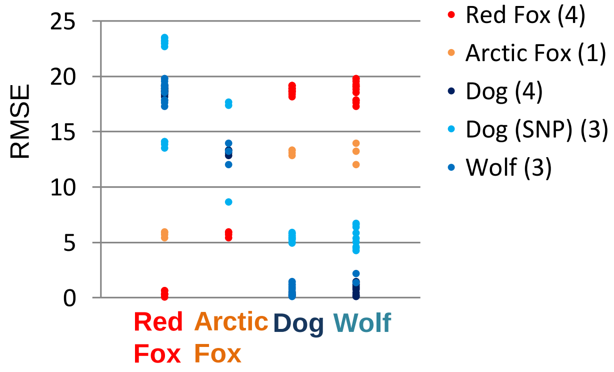

Normalisation: Leading range: 72.5 – 73.5 °C;
Trailing range: 78.0 – 79.0 °C

Cytb: HRM curves

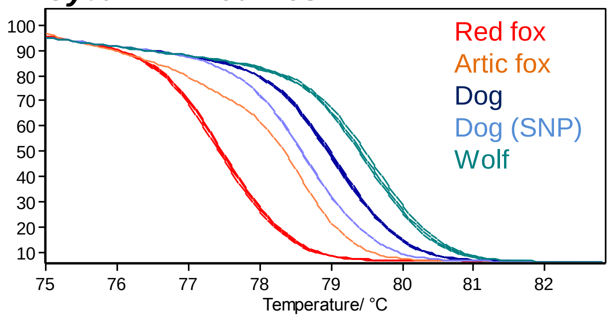

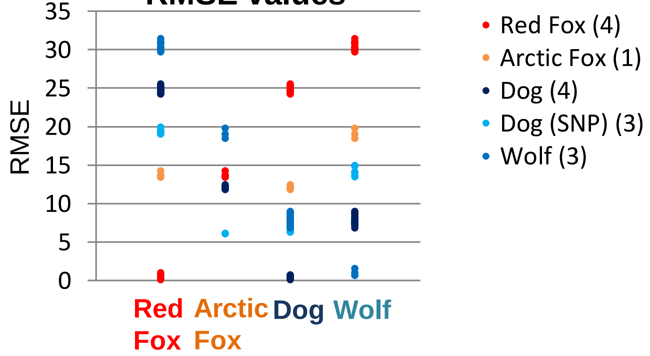

Normalisation: Leading range: 75.01 – 76.01 °C;
Trailing range: 81.81 – 82.81 °C

C Caprinae

12S rRNA: HRM curves

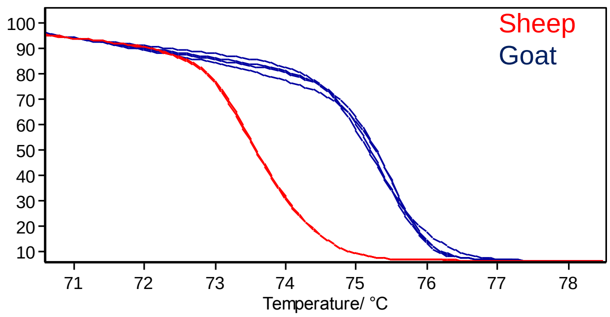

RMSE values

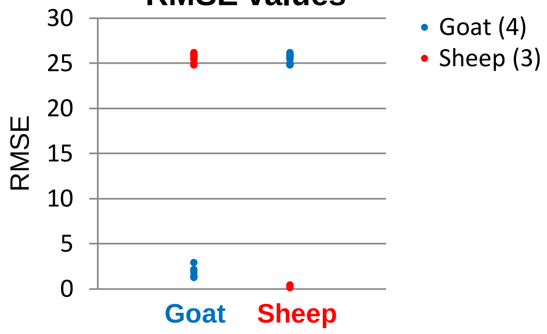

Normalisation: Leading range: 70.6 – 71.6 °C;
Trailing range: 77.5 – 78.5 °C

Cytb: HRM curves

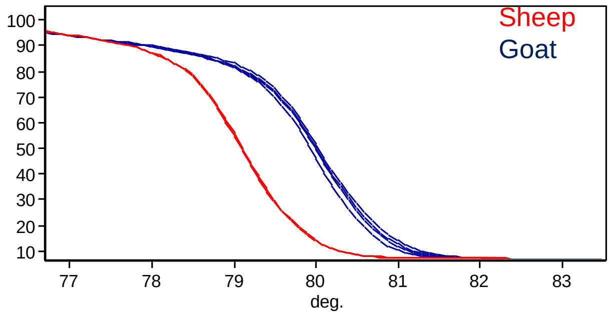

RMSE values

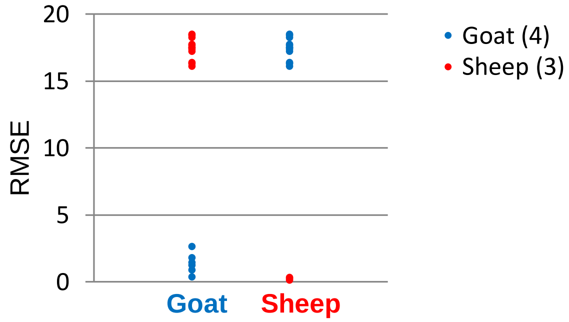

Normalisation: Leading range: 76.7 – 77.7 °C;
Trailing range: 82.5 – 83.5 °C

D Cervidae

12S rRNA: HRM curves

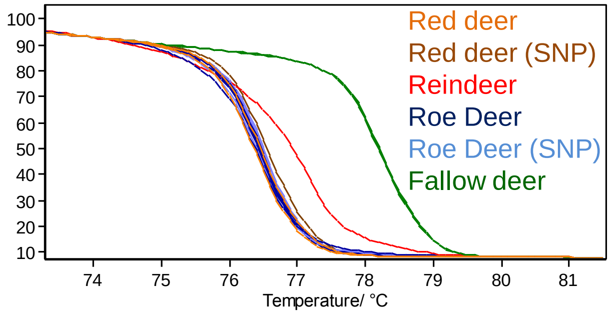

RMSE values

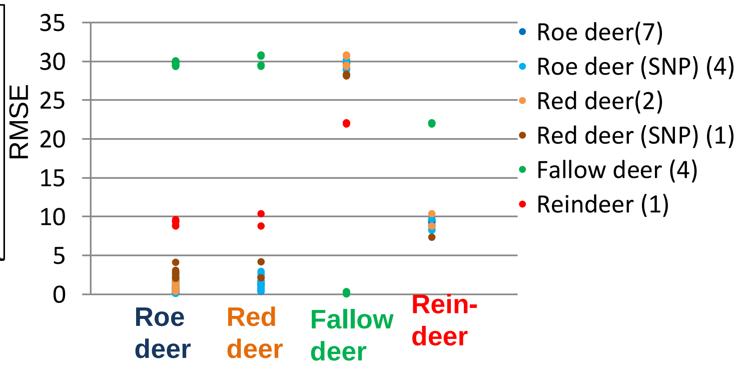

Normalisation: Leading range: 73.3 – 74.5 °C;
Trailing range: 80.1 – 81.3 °C

Cytb: HRM curves

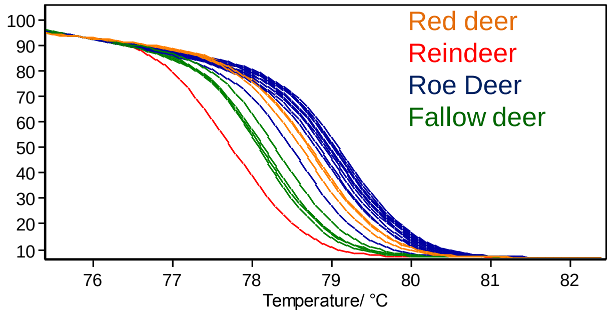

RMSE values

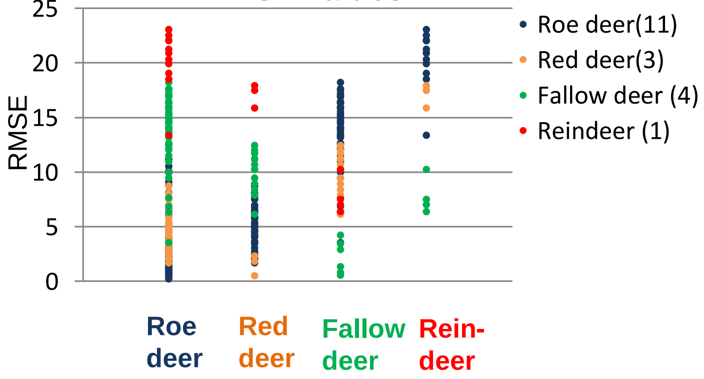

Normalisation: Leading range: 75.4 – 76.4 °C;
Trailing range: 81.4 – 82.4 °C

E Equidae

12S rRNA: HRM curves

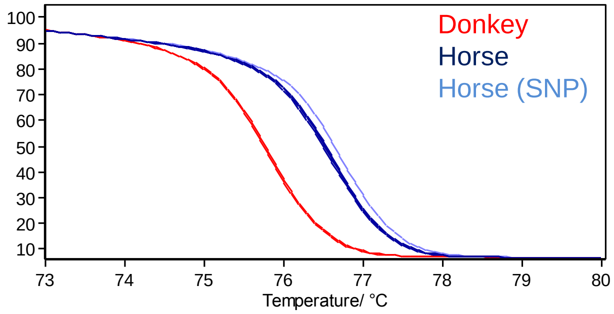

Normalisation: Leading range: 73.0 – 74.0 °C; Trailing range: 79.0 – 80.0 °C

RMSE values

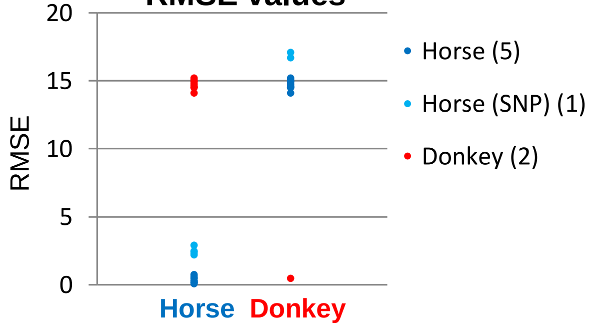

Cytb: HRM curves

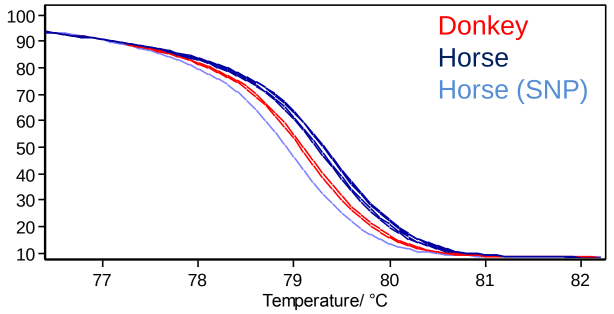

Normalisation: Leading range: 76.41 – 77.41 °C; Trailing range: 81.38 – 82.38 °C

RMSE values

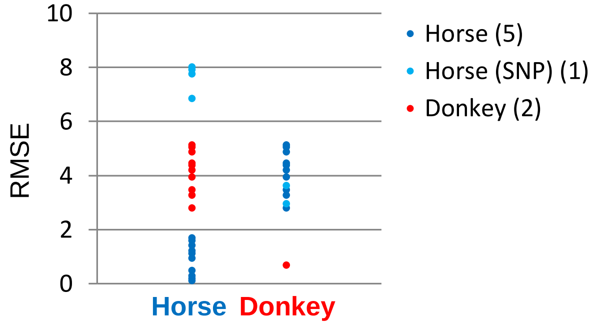

F Felidae

12S rRNA: HRM curves

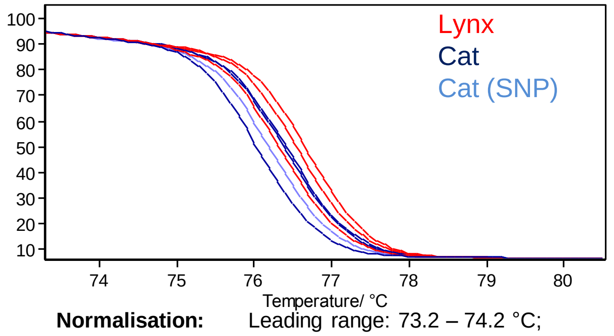

Normalisation: Leading range: 73.2 – 74.2 °C; Trailing range: 79.5 – 80.5 °C

RMSE values

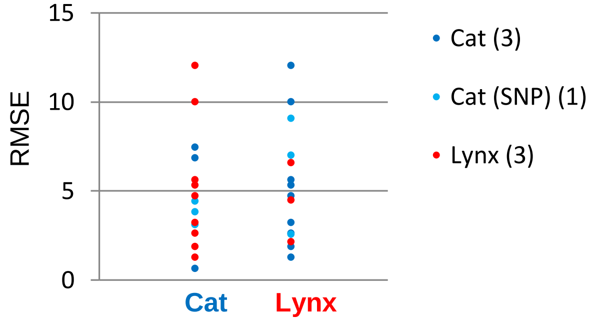

Cytb: HRM curves

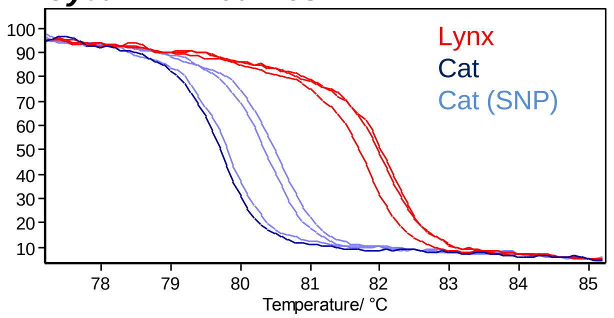

Normalisation: Leading range: 77.2 – 78.2 °C; Trailing range: 84.2 – 85.2 °C

RMSE values

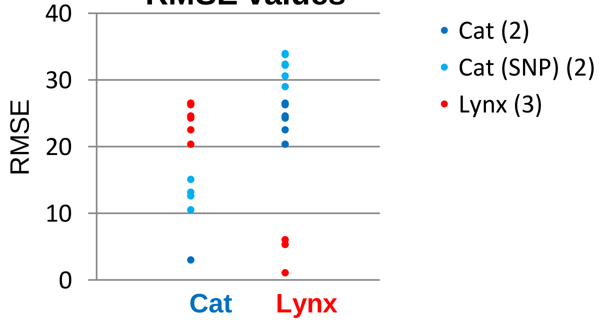

G Homo sapiens

12S rRNA: HRM curves

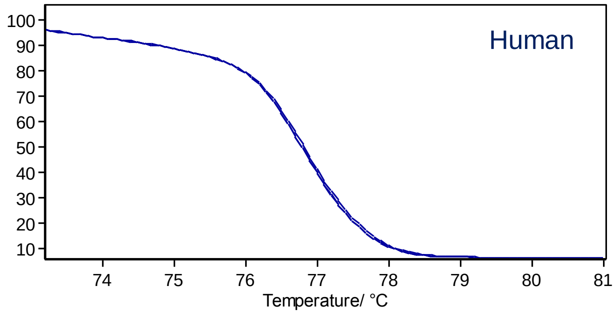

RMSE values

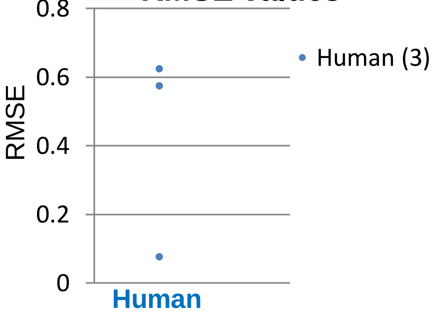

Normalisation: Leading range: 73.2 – 74.2 °C; Trailing range: 80.0 – 81.0 °C

Cytb: HRM curves

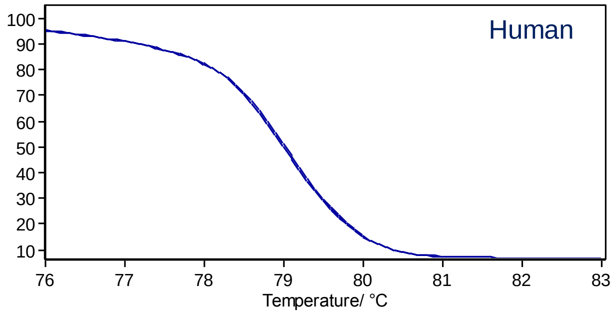

RMSE values

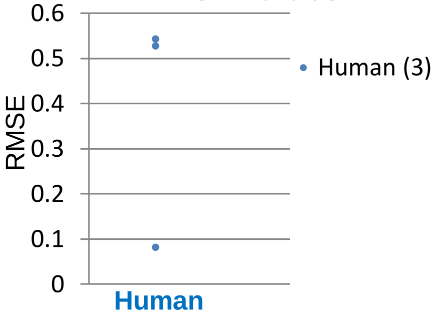

Normalisation: Leading range: 76.0 – 77.0 °C; Trailing range: 82.0 – 83.0 °C

H Leporidae

12S rRNA: HRM curves

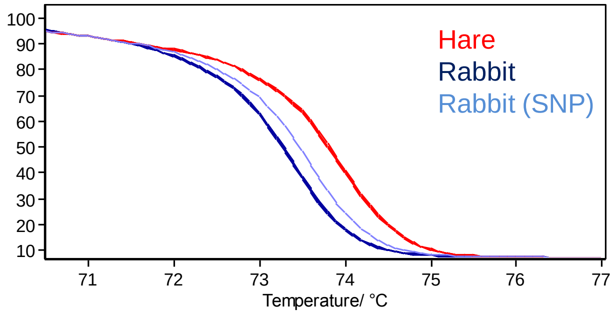

RMSE values

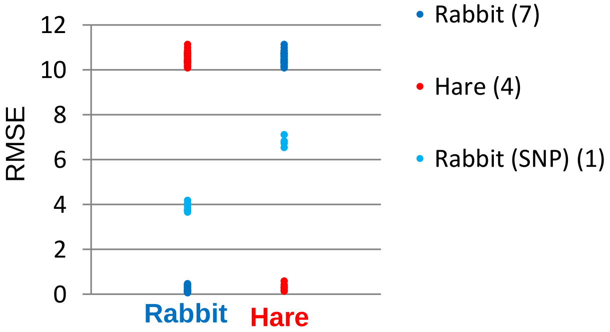

Normalisation: Leading range: 70.5 – 71.5 °C; Trailing range: 76.0 – 77.0 °C

Cytb: HRM curves

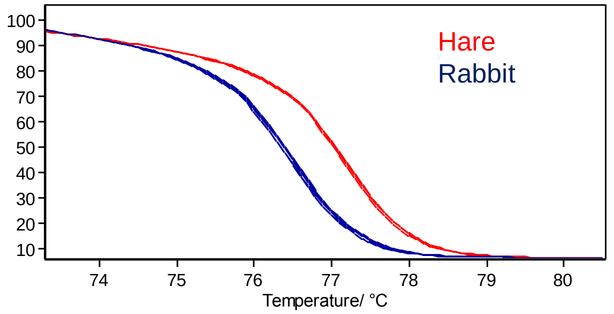

RMSE values

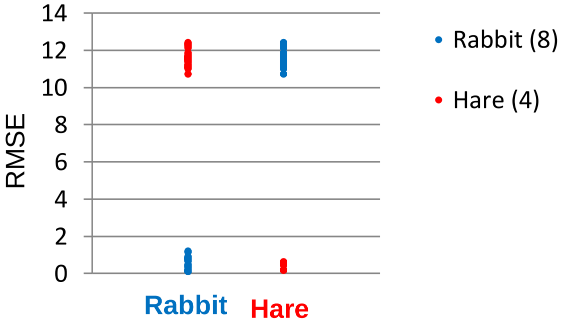

Normalisation: Leading range: 73.3 – 74.3 °C; Trailing range: 79.5 – 80.5 °C

I Mustelidae

12S rRNA: HRM curves

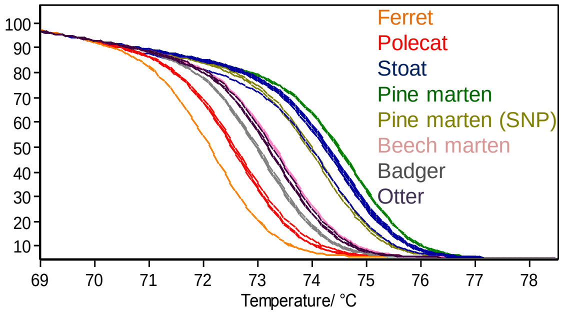

Normalisation: Leading range: 69.0 – 70.0 °C;
Trailing range: 77.5 – 78.5 °C

RMSE values

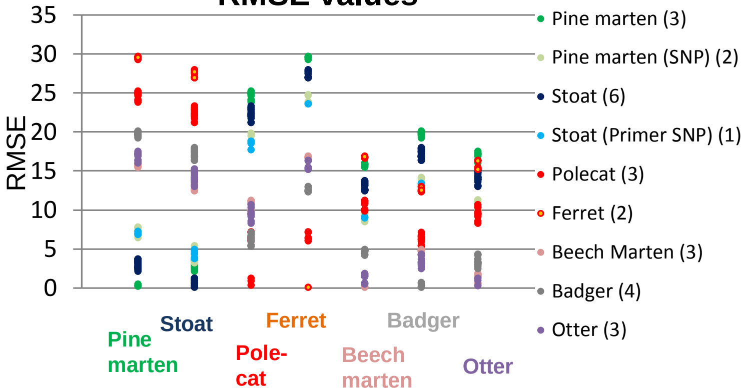

Cytb: HRM curves

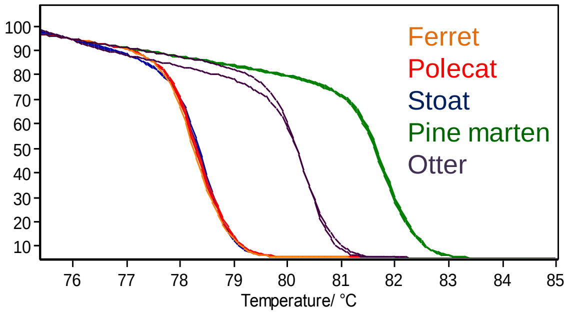

Normalisation: Leading range: 75.4 – 76.4 °C;
Trailing range: 84.0 – 85.0 °C

RMSE values

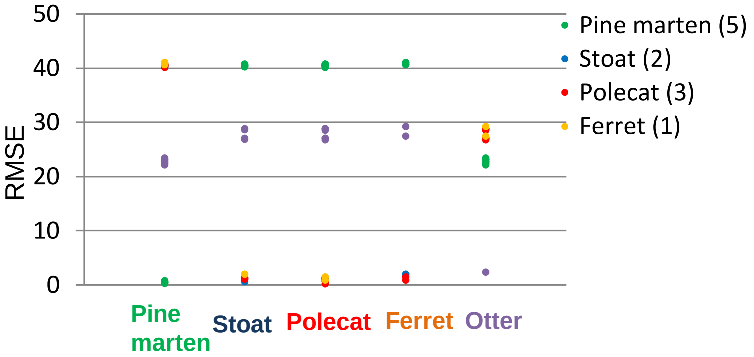

w/o (Ct>30): FRT96-98 (Beech marten), FRT99-102 (Badger), FRT84/FRT86/FRT88-90 (Stoat), FRT95 (Ferret), FRT103 (Otter)

J Phasianidae

12S rRNA: HRM curves

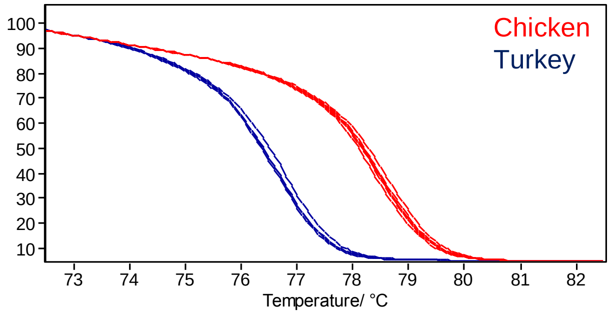

Normalisation: Leading range: 72.5 – 73.5 °C;
Trailing range: 81.5 – 82.5 °C

RMSE values

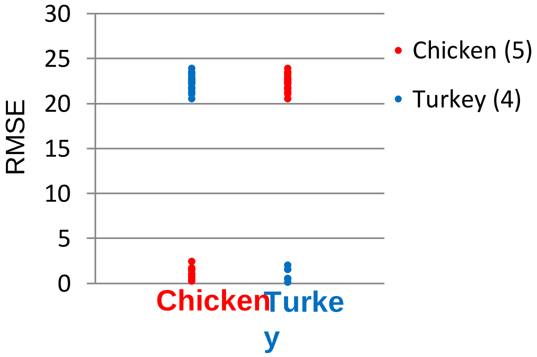

Cytb: HRM curves

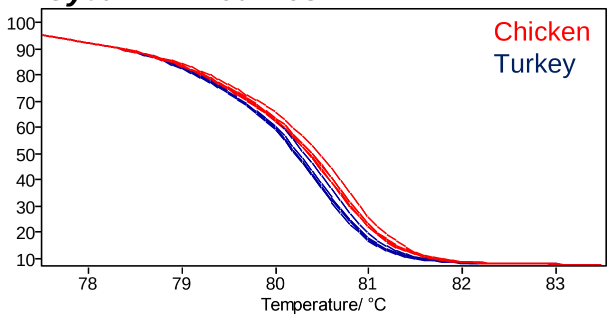

Normalisation: Leading range: 77.5 – 78.5 °C;
Trailing range: 82.5 – 83.5 °C

RMSE values

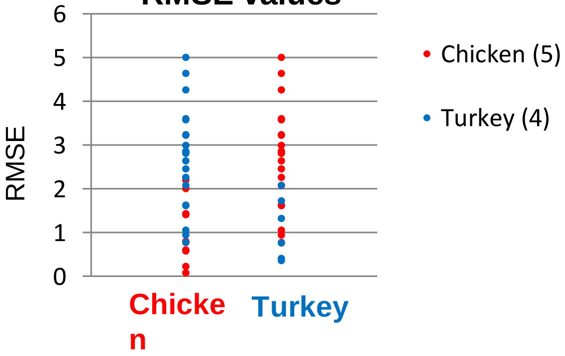

K Sus scrofa

12S rRNA: HRM curves

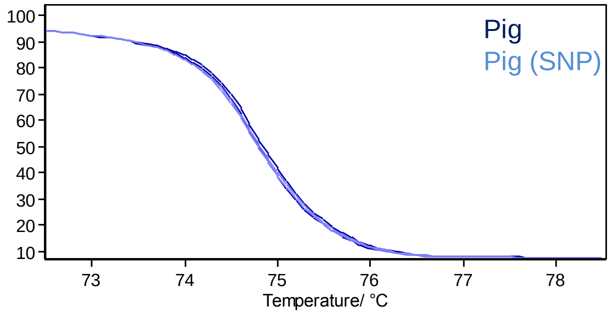

Normalisation: Leading range: 72.5 – 73.5 °C; Trailing range: 77.5 – 78.5 °C

RMSE values

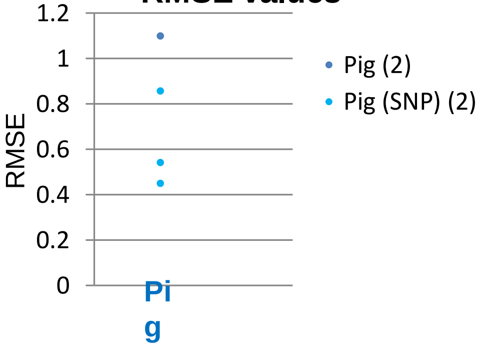

Cytb: HRM curves

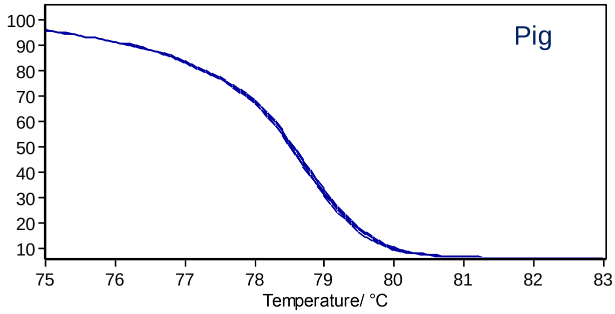

Normalisation: Leading range: 75.0 – 76.0 °C; Trailing range: 82.0 – 83.0 °C

RMSE values

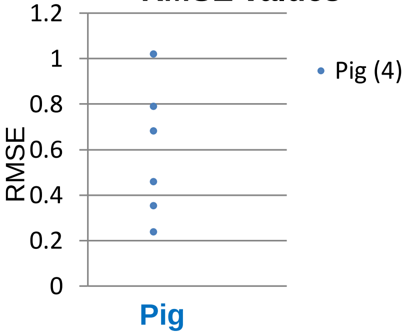

Supplement: S1 Fig — Normalized HRM curves and RMSE value ranges of animal-group-specific 12S rRNA and cytb analysis. (PDF) [file pone.0115575.s001.pdf]
