# Supplementary material for: High-Resolution Melting of 12S rRNA and Cytochrome b DNA Sequences for Discrimination of Species within Distinct European Animal Families
Source: PLoS One. 2014 Dec 22;9(12):e115575. doi: 10.1371/journal.pone.0115575 (PMC4274031; doi:10.1371/journal.pone.0115575)
Supplement: S2 Fig — HRM analysis with the universal 12S rRNA primer. The normalized HRM curves of two runs were merged into one figure. Additionally the curves were separated according to the animal groups. (PDF) [file pone.0115575.s002.pdf]

Figure S2

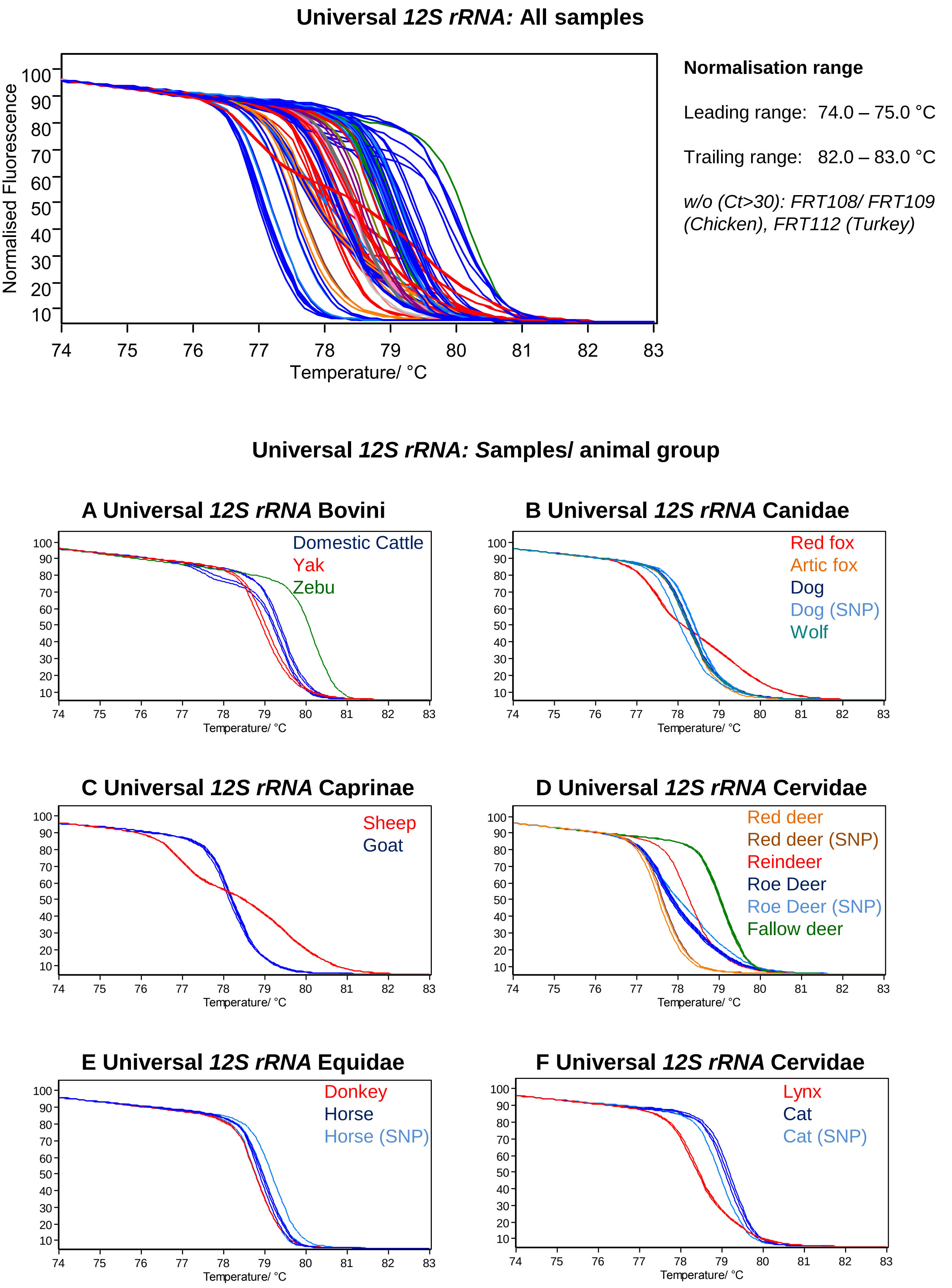
Universal 12S rRNA: All samples
Normalised Fluorescence
Temperature/ °C
Normalisation range
Leading range: 74.0 – 75.0 °C
Trailing range: 82.0 – 83.0 °C
w/o (Ct>30): FRT108/ FRT109 (Chicken), FRT112 (Turkey)
Universal 12S rRNA: Samples/ animal group
A Universal 12S rRNA Bovini
Domestic Cattle
Yak
Zebu
B Universal 12S rRNA Canidae
Red fox
Artic fox
Dog
Dog (SNP)
Wolf
C Universal 12S rRNA Caprinae
Sheep
Goat
D Universal 12S rRNA Cervidae
Red deer
Red deer (SNP)
Reindeer
Roe Deer
Roe Deer (SNP)
Fallow deer
E Universal 12S rRNA Equidae
Donkey
Horse
Horse (SNP)
F Universal 12S rRNA Cervidae
Lynx
Cat
Cat (SNP)

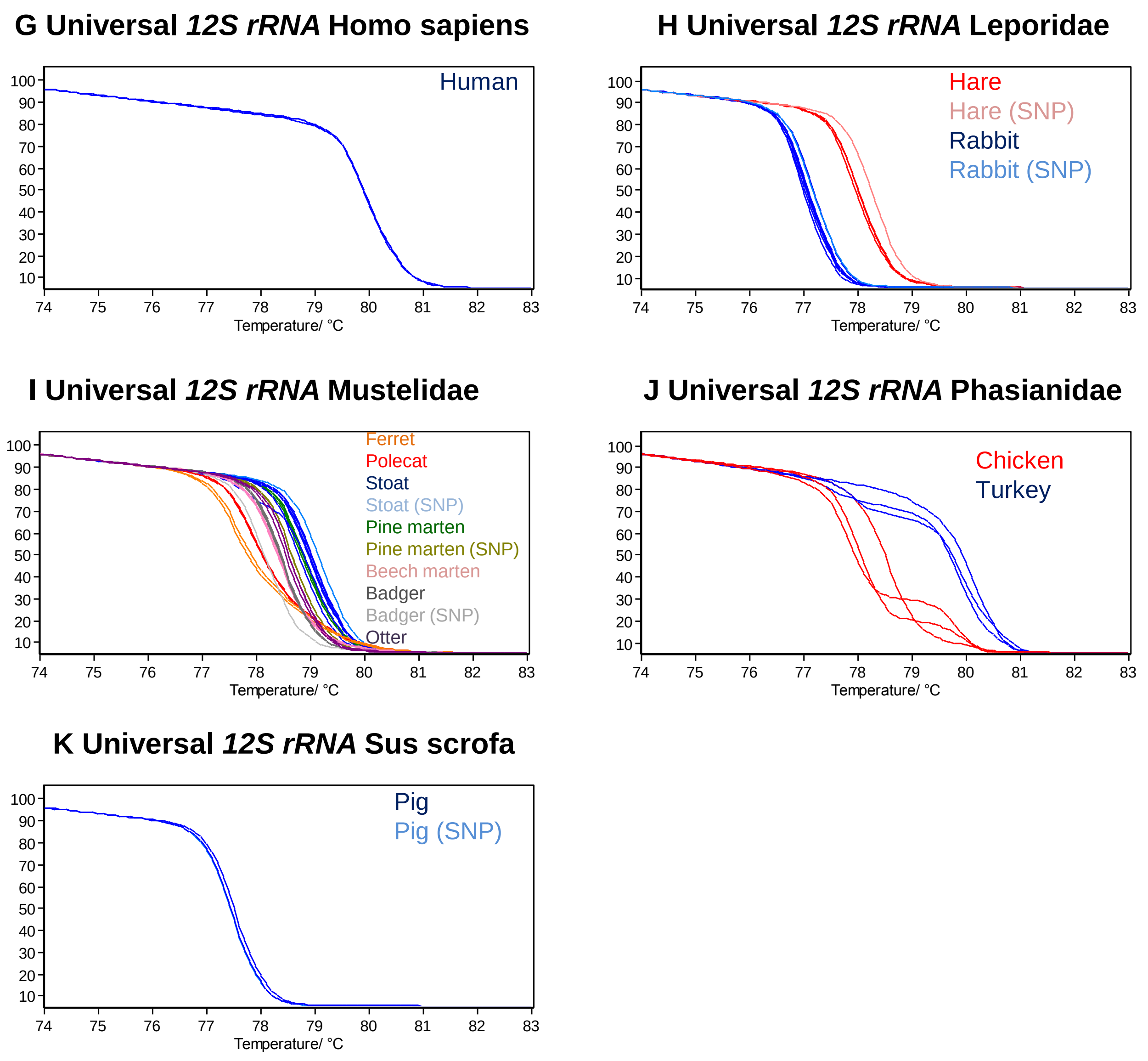

G Universal 12S rRNA Homo sapiens
Human
H Universal 12S rRNA Leporidae
Hare
Hare (SNP)
Rabbit
Rabbit (SNP)
I Universal 12S rRNA Mustelidae
Ferret
Polecat
Stoat
Stoat (SNP)
Pine marten
Pine marten (SNP)
Beech marten
Badger
Badger (SNP)
Otter
J Universal 12S rRNA Phasianidae
Chicken
Turkey
K Universal 12S rRNA Sus scrofa
Pig
Pig (SNP)
Temperature/ °C
