# Supplementary material for: High-Resolution Melting of 12S rRNA and Cytochrome b DNA Sequences for Discrimination of Species within Distinct European Animal Families
Source: PLoS One. 2014 Dec 22;9(12):e115575. doi: 10.1371/journal.pone.0115575 (PMC4274031; doi:10.1371/journal.pone.0115575)

Figure S3

Mixtures Rabbit/ Hare

Universal *12S rRNA*

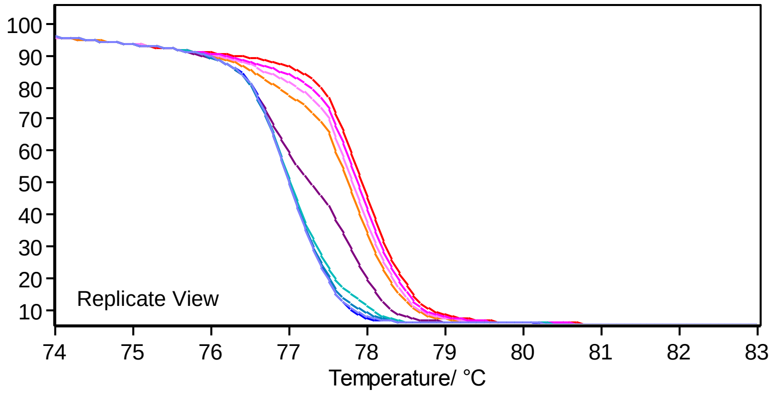

100% Rabbit
95% Rabbit / 5% Hare
90% Rabbit/ 10% Hare
80% Rabbit/ 20% Hare
50 % Rabbit / 50% Hare
20% Rabbit / 80% Hare
10% Rabbit/ 90% Hare
5% Rabbit/ 95% Hare
100% Hare

12S rRNA Leporidae

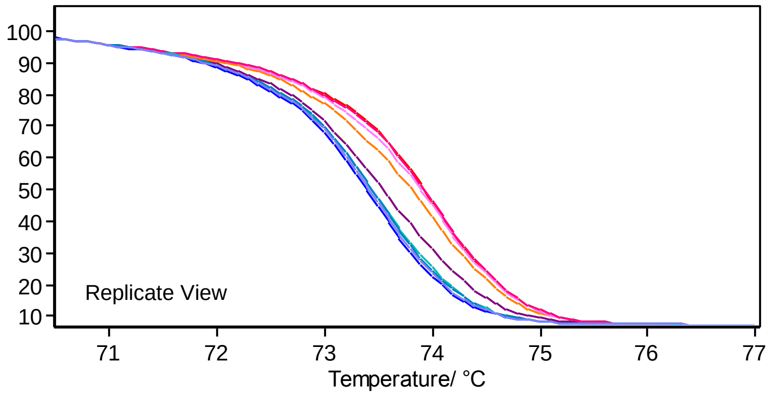

Cytb Leporidae

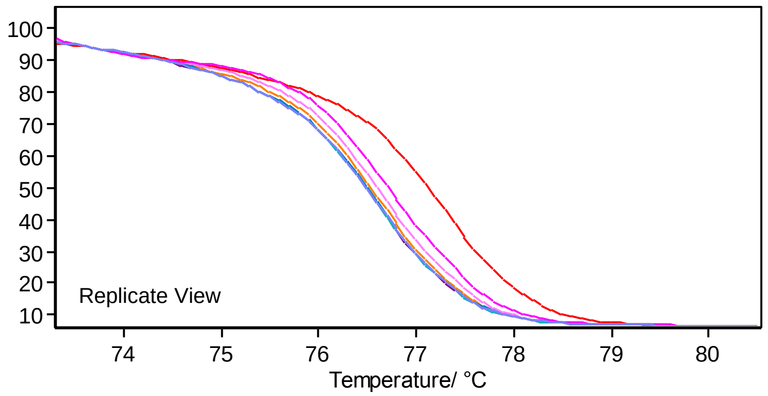

Mixtures Goat/ Sheep

Universal *12S rRNA*

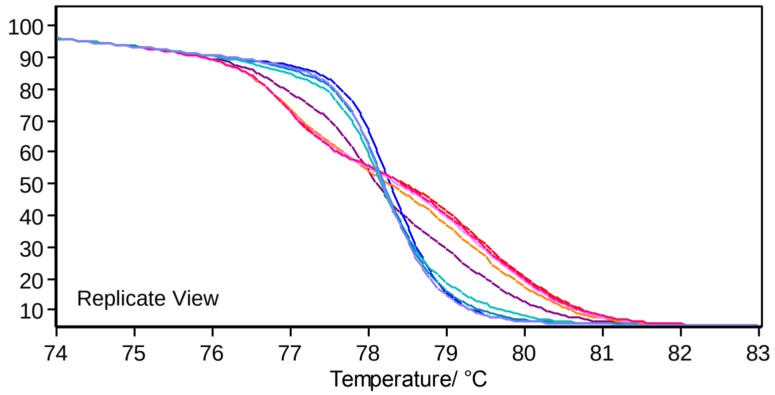

100% Goat
95% Goat / 5% Sheep
90% Goat/ 10% Sheep
80% Goat/ 20% Sheep
50 % Goat / 50% Sheep
20% Goat / 80% Sheep
10% Goat/ 90% Sheep
5% Goat/ 95% Sheep
100% Sheep

12S rRNA Caprinae

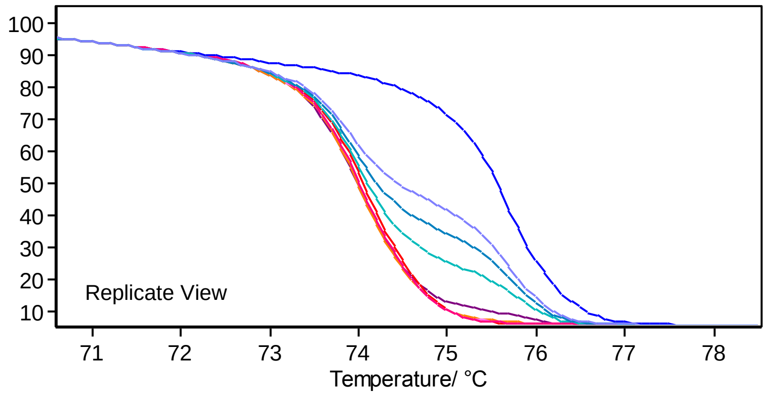

Cytb Caprinae

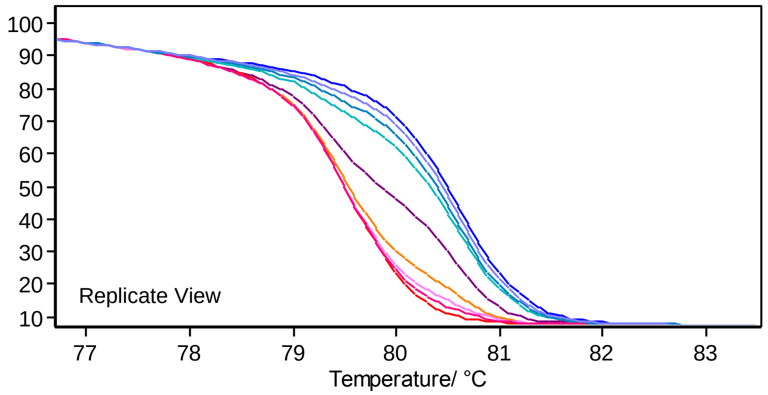

Mixtures Horse/ Donkey

Universal *12S rRNA*

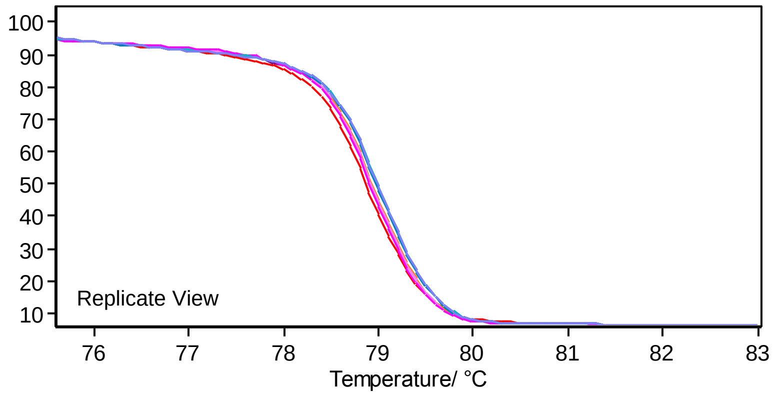

100% Horse
95% Horse / 5% Donkey
90% Horse/ 10% Donkey
80% Horse/ 20% Donkey
50 % Horse / 50% Donkey
20% Horse / 80% Donkey
10% Horse/ 90% Donkey
5% Horse/ 95% Donkey
100% Donkey

12S rRNA Equidae

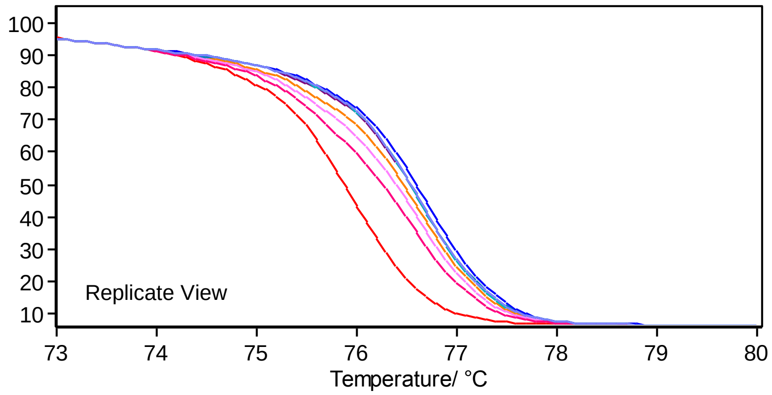

Cytb Equidae

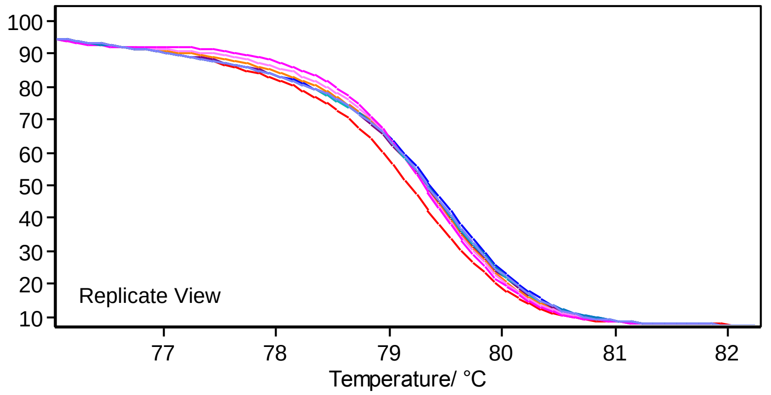

Supplement: S3 Fig — Normalized HRM curves for the group-specific12S rRNA, cytb and the universal 12S rRNA assays: analysis of different DNA mixtures. Normalization ranges correspond to the ranges used for each animal group. (PDF) [file pone.0115575.s003.pdf]
