# Supplementary material for: High-Resolution Melting of 12S rRNA and Cytochrome b DNA Sequences for Discrimination of Species within Distinct European Animal Families
Source: PLoS One. 2014 Dec 22;9(12):e115575. doi: 10.1371/journal.pone.0115575 (PMC4274031; doi:10.1371/journal.pone.0115575)
Supplement: S1 Table — Overview of the samples used for analysis. Species name, group assignment, accession numbers for the sequences, DNA amount, reference sequences for comparison and the differences to these are provided. (PDF) [file pone.0115575.s004.pdf]

Table S1

Animal family/tribe	Animal	Species	Sample ID	accession numbers (12S rRNA>200bp)	accession numbers (cytb >200bp))	Origin	Extraction method/ Elution buffer	DNA concentration (ng/µL)	used reference sequence	Mutation 12S rRNA	Mutation 12S rRNA primer sequence	Mutation cytb	Mutation cytb primer sequence	Mutation universal 12S additionally to specific 12S rRNA mutations	Mutation universal 12S rRNA primer sequence
Bovini (tribe)	Domestic cattle	*Bos primigenius taurus*	FRT1	KM224229	-	blood	Q-elution buffer	4.95	V00654						
Bovini (tribe)	Domestic cattle	*Bos primigenius taurus*	FRT2	KM224230	-	blood	Q-elution buffer	22.2	V00654						
Bovini (tribe)	Domestic cattle	*Bos primigenius taurus*	FRT3	KM224231	-	blood	Q-elution buffer	19.85	V00654						
Bovini (tribe)	Domestic cattle	*Bos primigenius taurus*	FRT4	KM224232	-	blood	Q-elution buffer	4.89	V00654						
Bovini (tribe)	Yak	*Bos mutus*	FRT5	KM224233	-	muscle	PhChl-H2O	68.8	no reference						
Bovini (tribe)	Yak	*Bos mutus*	FRT6	KM224234	-	buccal cells	Q-H2O	1.74	no reference						
Bovini (tribe)	Zebu	*Bos primigenius indicus*	FRT7	KM224235	-	muscle	PhChl-H2O	26.2	NC_005971		1 MM F			726T>C	
Canidae	red fox	*Vulpes vulpes*	FRT8	KM224236	KM224343	muscle	Q-H2O	3.02	NC_008434		1MM F				
Canidae	red fox	*Vulpes vulpes*	FRT9	KM224237	KM224344	muscle	Q-H2O	8.11	NC_008434		1MM F				
Canidae	red fox	*Vulpes vulpes*	FRT10	KM224238	KM224345	muscle	Q-H2O	3.12	NC_008434		1MM F				
Canidae	red fox	*Vulpes vulpes*	FRT11	KM224239	KM224346	muscle	Q-H2O	4.88	NC_008434		1MM F				
Canidae	Artic fox	*Vulpes lagopus*	FRT12	KM224240	KM224347	muscle	Q-H2O	6.36	12S rRNA: GU174607; cytb: AY598511	308G>A	2MM F, 1MM R	477T>C; 478C>T	6 MMF; 1 MMR		
Canidae	Dog	*Canis lupus familiaris*	FRT13	KM224241	KM224348	muscle	Q-elution buffer	54.6	NC_002008	381T>A; 445A>G		14671G>A			
Canidae	Dog	*Canis lupus familiaris*	FRT14	KM224242	KM224349	muscle	Q-H2O	2.16	NC_002008	377C>T					
Canidae	Dog	*Canis lupus familiaris*	FRT15	KM224243	KM224350	muscle	Q-H2O	0.49	NC_002008						
Canidae	Dog	*Canis lupus familiaris*	FRT16	KM224244	KM224351	muscle	Q-H2O	0.26	NC_002008						
Canidae	Dog	*Canis lupus familiaris*	FRT17	KM224245	KM224352	muscle	Q-H2O	24.5	NC_002008	381T>A; 445A>G		14671G>A			
Canidae	Dog	*Canis lupus familiaris*	FRT18	KM224246	KM224353	muscle	Q-H2O	38.25	NC_002008						
Canidae	Dog	*Canis lupus familiaris*	FRT19	KM224247	KM224354	muscle	Q-H2O	3.5	NC_002008						
Canidae	Wolf	*Canis lupus*	FRT20	KM224248	KM224355	muscle	Q-H2O	0.08	NC_002008			14665A>G			
Canidae	Wolf	*Canis lupus*	FRT21	KM224249	KM224356	muscle	Q-H2O	0.21	NC_002008			14665A>G			
Canidae	Wolf	*Canis lupus*	FRT22	KM224250	KM224357	muscle	Q-H2O	3.78	NC_002008			14665A>G			
Caprinae (Sub-family)	Goat	*Capra aegagrus hircus*	FRT23	-	KM224358	blood	Q-elution buffer	0.2	GU295658		1MM R				
Caprinae (Sub-family)	Goat	*Capra aegagrus hircus*	FRT24	-	KM224359	blood	Q-elution buffer	0.09	GU295658		1MM R				
Caprinae (Sub-family)	Goat	*Capra aegagrus hircus*	FRT25	-	KM224360	blood	Q-elution buffer	0.1	GU295658		1MM R				
Caprinae (Sub-family)	Goat	*Capra aegagrus hircus*	FRT26	-	KM224361	muscle	Q-H2O	8.28	GU295658		1MM R				
Caprinae (Sub-family)	Sheep	*Ovis (orientalis) aries*	FRT27	KM224251	KM224362	muscle	Q-H2O	13.95	NC_001941				1MM		
Caprinae (Sub-family)	Sheep	*Ovis (orientalis) aries*	FRT28	KM224252	KM224363	muscle	Q-H2O	14.1	NC_001941				1MM		
Caprinae (Sub-family)	Sheep	*Ovis (orientalis) aries*	FRT29	KM224253	KM224364	muscle	Q-H2O	4.4	NC_001941				1MM		
Cervidae	Roe deer	*Capreolus capreolus*	FRT30	KM224254	-	muscle	PhChl-H2O	118	NC_020684		1MM R	14702C>T	1MMF, 4MMR		
Cervidae	Roe deer	*Capreolus capreolus*	FRT31	KM224255	-	muscle	PhChl-H2O	110	NC_020684		1MM R	14702C>T	1MMF, 4MMR		
Cervidae	Roe deer	*Capreolus capreolus*	FRT32	KM224256	-	muscle	PhChl-H2O	114	NC_020684	374T>C;434 G>A	1MM R	14702C>T	1MMF, 4MMR		
Cervidae	Roe deer	*Capreolus capreolus*	FRT33	KM224257	-	muscle	Q-elution buffer	46.2	NC_020684	374T>C;434 G>A	1MM R	14702C>T	1MMF, 4MMR		
Cervidae	Roe deer	*Capreolus capreolus*	FRT34	KM224258	-	muscle	Q-H2O	28.4	NC_020684		1MM R	14702C>T	1MMF, 4MMR		
Cervidae	Roe deer	*Capreolus capreolus*	FRT35	KM224259	-	muscle	Q-H2O	12.6	NC_020684	382del	1MM R	14702C>T	1MMF, 4MMR		
Cervidae	Roe deer	*Capreolus capreolus*	FRT36	KM224260	-	muscle	Q-H2O	8.34	NC_020684		1MM R	14702C>T	1MMF, 4MMR		
Cervidae	Roe deer	*Capreolus capreolus*	FRT37	KM224261	-	muscle	Q-H2O	9.13	NC_020684		1MM R	14702C>T	1MMF, 4MMR		
Cervidae	Roe deer	*Capreolus capreolus*	FRT38	KM224262	-	muscle	Q-H2O	30.1	NC_020684		1MM R	14702C>T	1MMF, 4MMR		
Cervidae	Roe deer	*Capreolus capreolus*	FRT39	KM224263	KM224365	muscle	Q-H2O	0.46	NC_020684	382del	1MM R	14702C>T	1MMF, 4MMR		
Cervidae	Roe deer	*Capreolus capreolus*	FRT40	KM224264	-	muscle	Q-H2O	0.74	NC_020684		1MM R	14702C>T	1MMF, 4MMR		
Cervidae	Red deer	*Cervus elaphus*	FRT41	KM224265	KM224366	muscle	PhChl-H2O	25.03	NC_007704	396C>T;423T>C	1MM F		1MM F; 1MM R		1MM R
Cervidae	Red deer	*Cervus elaphus*	FRT42	KM224266	KM224367	blood	Q-H2O	1.44	NC_007704		1MM F				1MM R
Cervidae	Red deer	*Cervus elaphus*	FRT43	KM224267	KM224368	muscle	Q-H2O	21.2	NC_007704		1MM F				1MM R
Cervidae	Fallow deer	*Dama dama*	FRT44	KM224268	KM224369	muscle	PhChl-H2O	64	NC_020700		1MM F		1MMF; 4MM R		1MM F; 1MM R
Cervidae	Fallow deer	*Dama dama*	FRT45	KM224269	KM224370	muscle	Q-H2O	5.69	NC_020700		1MM F		1MMF; 4MM R		1MM F; 1MM R
Cervidae	Fallow deer	*Dama dama*	FRT46	KM224270	-	muscle	Q-H2O	1.19	NC_020700		1MM F		1MMF; 4MM R		1MM F; 1MM R
Cervidae	Fallow deer	*Dama dama*	FRT47	KM224271	-	muscle	Q-H2O	4.54	NC_020700		1MM F		1MMF; 4MM R		1MM F; 1MM R
Cervidae	Reindeer	*Rangifer tarandus*	FRT48	KM224272	KM224371	muscle	Q-H2O	3.78	NC_007703		2MM F; 2MM R		3MMF; 3MMR		1MM R
Equidae	Horse	*Equus ferus caballus*	FRT49	KM224273	KM224372	muscle	Q-elution buffer	57.9	X79547	386A>G;415C>T	1MM F		1MM F	356T>C;357.1C; 381C>T	
Equidae	Horse	*Equus ferus caballus*	FRT50	KM224274	KM224373	muscle	Q-H2O	1.73	X79547			14673G>A		356T>C;357.1C	
Equidae	Horse	*Equus ferus caballus*	FRT51	KM224275	KM224374	muscle	Q-H2O	0.21	X79547					356T>C;357.1C	
Equidae	Horse	*Equus ferus caballus*	FRT52	KM224276	KM224375	muscle	Puregene-1xTE buffer	47.4	X79547					341A>G; 356T>C;357.1C	
Equidae	Horse	*Equus ferus caballus*	FRT53	KM224277	KM224376	muscle	Puregene-1xTE buffer	27.7	X79547					356T>C;357.1C	
Equidae	Horse	*Equus ferus caballus*	FRT54	KM224278	KM224377	muscle	Puregene-1xTE buffer	33.5	X79547					356T>C;357.1C	
Equidae	Donkey	*Equus asinus asinus*	FRT55	KM224279	-	buccal cells	Q-H2O	1.54	NC_001788				1MM F		1MM R
Equidae	Donkey	*Equus asinus asinus*	FRT56	KM224280	-	bucal cells	Q-H2O	2.95	NC_001788				1MM F		1MM R
Felidae	Cat	*Felis silvestris catus*	FRT57	KM224281	KM224378	muscle	Puregene-1xTE buffer	18.3	NC_001700			15577G>C, 15590T>C	1 MM R		1MM F
Felidae	Cat	*Felis silvestris catus*	FRT58	KM224282	KM224379	muscle	Puregene-1xTE buffer	24.7	NC_001700			15577G>C, 15590T>C	1 MM R		1MM F
Felidae	Cat	*Felis silvestris catus*	FRT59	KM224283	KM224380	muscle	Puregene-1xTE buffer	12.45	NC_001700	1276G>A		15577G>C, 15578C>T	1 MM R		1MM F
Felidae	Cat	*Felis silvestris catus*	FRT60	KM224284	-	muscle	Puregene-1xTE buffer	50.4	NC_001700			15577G>C	2 MM R		1MM F
Felidae	Lynx	*Lynx lynx*	FRT61	KM224285	-	muscle or liver	Puregene-1xTE buffer	15.3	no reference		1MM F				
Felidae	Lynx	*Lynx lynx*	FRT62	KM224286	-	muscle or liver	Puregene-1xTE buffer	12.95	no reference		1MM F		1 MM R		
Felidae	Lynx	*Lynx lynx*	FRT63	KM224287	-	muscle or liver	Puregene-1xTE buffer	16.4	no reference		1MM F				
Homo sapiens	Human	*Homo sapiens*	FRT64	KM224288	KM224381	buccal cells	Q-H2O	5.56	NC_012920						
Homo sapiens	Human	*Homo sapiens*	FRT65	KM224289	KM224382	buccal cells	Q-H2O	4.21	NC_012920						
Homo sapiens	Human	*Homo sapiens*	FRT66	KM224290	KM224383	buccal cells	Q-H2O	4.14	NC_012920						
Leporidae	Rabbit	*Oryctolagus cuniculus*	FRT67	KM224291	KM224384	muscle	Q-H2O	4.41	NC_001913	414G>A			1MM F, 1MM R		
Leporidae	Rabbit	*Oryctolagus cuniculus*	FRT68	KM224292	KM224385	muscle	Q-H2O	2.79	NC_001913	414G>A			1MM F, 1MM R		

Leporidae	Rabbit	*Oryctolagus cuniculus*	FRT69	KM224293	KM224386	muscle	Q-H2O	5.62	NC_001913				1MM F, 1MM R		
Leporidae	Rabbit	*Oryctolagus cuniculus*	FRT70	KM224294	KM224387	muscle	Q-H2O	16.75	NC_001913	414G>A			1MM F, 1MM R		
Leporidae	Rabbit	*Oryctolagus cuniculus*	FRT71	KM224295	KM224388	muscle	Q-H2O	27.8	NC_001913	414G>A			1MM F, 1MM R		
Leporidae	Rabbit	*Oryctolagus cuniculus*	FRT72	KM224296	KM224389	muscle	Q-H2O	15.5	NC_001913	414G>A			1MM F, 1MM R		
Leporidae	Rabbit	*Oryctolagus cuniculus*	FRT73	KM224297	KM224390	muscle	Q-H2O	10.8	NC_001913	414G>A			1MM F, 1MM R		
Leporidae	Rabbit	*Oryctolagus cuniculus*	FRT74	KM224298	KM224391	muscle	Q-H2O	19.5	NC_001913	414G>A			1MM F, 1MM R		
Leporidae	Hare	*Lepus europaeus*	FRT75	KM224299	KM224392	muscle	PhChl-H2O	108	NC_004028				3MM R	350T>A	1MM R
Leporidae	Hare	*Lepus europaeus*	FRT76	KM224300	KM224393	muscle	Q-H2O	7.03	NC_004028				3MM R	350T>A	1MM R
Leporidae	Hare	*Lepus europaeus*	FRT77	KM224301	KM224394	muscle	Q-H2O	22.5	NC_004028				3MM R	350T>A	1MM R
Leporidae	Hare	*Lepus europaeus*	FRT78	KM224302	KM224395	muscle	Q-H2O	0.19	NC_004028				3MM R	350T>A; 476T>C	1MM R
Mustelidae	Pine marten	*Martes martes*	FRT79	KM224303	KM224396	muscle or liver	Puregene-1xTE buffer	33	NC_021749	383C>T	1 MM F		1MM F; 1MM R		2MM R
Mustelidae	Pine marten	*Martes martes*	FRT80	KM224304	KM224397	muscle or liver	Puregene-1xTE buffer	25.6	NC_021749		1 MM F		1MM F; 1MM R		2MM R
Mustelidae	Pine marten	*Martes martes*	FRT81	KM224305	KM224398	muscle or liver	Puregene-1xTE buffer	24.2	NC_021749		1 MM F		1MM F; 1MM R		2MM R
Mustelidae	Pine marten	*Martes martes*	FRT82	KM224306	KM224399	muscle or liver	Puregene-1xTE buffer	12.2	NC_021749	383C>T	1 MM F		1MM F; 1MM R		2MM R
Mustelidae	Pine marten	*Martes martes*	FRT83	KM224307	KM224400	muscle or liver	Puregene-1xTE buffer	19.65	NC_021749				1MM F; 1MM R		2MM R
Mustelidae	Stoat	*Mustela erminea*	FRT84	KM224308	-	muscle or liver	Puregene-1xTE buffer	38.1	12S rRNA: AB119066, cytb: AB026101			523T>C	1MM R	393T>C	2MM R
Mustelidae	Stoat	*Mustela erminea*	FRT85	KM224309	KM224401	muscle or liver	Puregene-1xTE buffer	30.6	12S rRNA: AB119066, cytb: AB026101				1MM R		2MM R
Mustelidae	Stoat	*Mustela erminea*	FRT86	KM224310	KM224402	muscle or liver	Puregene-1xTE buffer	78.5	12S rRNA: AB119066, cytb: AB026101				1MM R		2MM R
Mustelidae	Stoat	*Mustela erminea*	FRT87	KM224311	-	muscle or liver	Puregene-1xTE buffer	99.8	12S rRNA: AB119066, cytb: AB026101		1MMF		1MM R	306G>A	2MM R
Mustelidae	Stoat	*Mustela erminea*	FRT88	KM224312	KM224403	muscle or liver	Puregene-1xTE buffer	12.4	12S rRNA: AB119066, cytb: AB026101				1MM R		2MM R
Mustelidae	Stoat	*Mustela erminea*	FRT89	KM224313	KM224404	muscle or liver	Puregene-1xTE buffer	106	12S rRNA: AB119066, cytb: AB026101				1MM R		2MM R
Mustelidae	Stoat	*Mustela erminea*	FRT90	KM224314	KM224405	muscle or liver	Puregene-1xTE buffer	19.5	12S rRNA: AB119066, cytb: AB026101				1MM R		2MM R
Mustelidae	European polecat	*Mustela putorius*	FRT91	KM224315	KM224406	muscle or liver	Puregene-1xTE buffer	35	NC_020638	389T>C	1MMR				1MMR
Mustelidae	European polecat	*Mustela putorius*	FRT92	KM224316	KM224407	muscle or liver	Puregene-1xTE buffer	20.35	NC_020638	389T>C	1MMR				1MMR
Mustelidae	European polecat	*Mustela putorius*	FRT93	KM224317	KM224408	muscle or liver	Puregene-1xTE buffer	25.1	NC_020638	389T>C	1MMR				1MMR
Mustelidae	Ferret	*Mustela putorius furo*	FRT94	KM224318	KM224409	muscle	Q-H2O	38.4	NC_020638		1MMR				1MMR
Mustelidae	Ferret	*Mustela putorius furo*	FRT95	KM224319	KM224410	muscle	Puregene-1xTE buffer	16.75	NC_020638		1MMR				1MMR
Mustelidae	Beech marten	*Martes foina*	FRT96	KM224320	KM224411	muscle or liver	Puregene-1xTE buffer	45.2	NC_020643	384 C>T		14668G>A;14698A>G	2MM F, 1MMR	357T>C	2MM R
Mustelidae	Beech marten	*Martes foina*	FRT97	KM224321	KM224412	muscle or liver	Puregene-1xTE buffer	21.5	NC_020643	384 C>T		14668G>A;14698A>G	2MM F, 1MMR	357T>C	2MM R
Mustelidae	Beech marten	*Martes foina*	FRT98	KM224322	KM224413	muscle or liver	Puregene-1xTE buffer	32.27	NC_020643	384 C>T		14668G>A;14698A>G	2MM F, 1MMR	357T>C	2MM R
Mustelidae	European badger	*Meles meles*	FRT99	KM224323	KM224414	muscle or liver	Puregene-1xTE buffer	27	NC_011125				2MM F, 1MMR	345C>T;469T>C	1MMR
Mustelidae	European badger	*Meles meles*	FRT100	KM224324	KM224415	muscle or liver	Puregene-1xTE buffer	36.7	NC_011125				2MM F, 1MMR	345C>T;469T>C	1MMR
Mustelidae	European badger	*Meles meles*	FRT101	KM224325	KM224416	muscle or liver	Puregene-1xTE buffer	26.8	NC_011125				2MM F, 1MMR	345C>T	1MMR
Mustelidae	European badger	*Meles meles*	FRT102	KM224326	KM224417	muscle or liver	Puregene-1xTE buffer	103	NC_011125			14682T>C	2MM F, 1MMR	345C>T;469T>C	1MMR
Mustelidae	European otter	*Lutra lutra*	FRT103	KM224327	KM224418	muscle or liver	Puregene-1xTE buffer	45.2	NC_011358			14673G>A; 14712T>C	2MM F		2MM R
Mustelidae	European otter	*Lutra lutra*	FRT104	KM224328	KM224419	muscle or liver	Puregene-1xTE buffer	58.5	NC_011358			14673G>A; 14712T>C	2MM F		2MM R
Mustelidae	European otter	*Lutra lutra*	FRT105	KM224329	KM224420	muscle or liver	Puregene-1xTE buffer	9.35	NC_011358			14673G>A; 14712T>C	2MM F		2MM R
Phasianidae	Chicken	*Gallus gallus domesticus*	FRT106	KM224330	KM224421	muscle	PhChl-H2O	44	NC_001323		1MMR		1MMR		3MM F; 1MMR
Phasianidae	Chicken	*Gallus gallus domesticus*	FRT107	KM224331	KM224422	muscle	Q-H2O	5.35	NC_001323		1MMR		1MMR		3MM F; 1MMR
Phasianidae	Chicken	*Gallus gallus domesticus*	FRT108	KM224332	KM224423	muscle	Q-H2O	10.2	NC_001323		1MMR		1MMR		3MM F; 1MMR
Phasianidae	Chicken	*Gallus gallus domesticus*	FRT109	KM224333	KM224424	muscle	Q-H2O	10.1	NC_001323		1MMR		1MMR		3MM F; 1MMR
Phasianidae	Chicken	*Gallus gallus domesticus*	FRT110	KM224334	KM224425	muscle	Q-H2O	8.53	NC_001323		1MMR		1MMR		3MM F; 1MMR
Phasianidae	Turkey	*Meleagris gallopavo*	FRT111	KM224335	KM224426	muscle	Q-elution buffer	1	NC_010195		1MMF, MMR				3MM F; 4MMR
Phasianidae	Turkey	*Meleagris gallopavo*	FRT112	KM224336	KM224427	muscle	Q-H2O	1.36	NC_010195		1MMF, MMR				3MM F; 4MMR
Phasianidae	Turkey	*Meleagris gallopavo*	FRT113	KM224337	KM224428	muscle	Q-H2O	4.08	NC_010195		1MMF, MMR				3MM F; 4MMR
Phasianidae	Turkey	*Meleagris gallopavo*	FRT114	KM224338	KM224429	muscle	Q-H2O	5.18	NC_010195		1MMF, MMR				3MM F; 4MMR
Sus scrofa domestica	Pig	*Sus scrofa domestica*	FRT115	KM224339	KM224430	blood	Q-elution buffer	4.43	NC_012095						1MM R
Sus scrofa domestica	Pig	*Sus scrofa domestica*	FRT116	KM224340	KM224431	muscle	Q-H2O	9.07	NC_012095						1MM R
Sus scrofa domestica	Pig	*Sus scrofa domestica*	FRT117	KM224341	KM224432	muscle	Q-H2O	6.46	NC_012095	383.1A					1MM R
Sus scrofa domestica	Pig	*Sus scrofa domestica*	FRT118	KM224342	KM224433	muscle	Q-H2O	35.5	NC_012095	383.1A					1MM R

PhChl- Phenol/chloroform
Q-Qiagen Mini Kit
1xTE buffer with residual NaCl amount
