# Supplementary material for: High-Resolution Melting of 12S rRNA and Cytochrome b DNA Sequences for Discrimination of Species within Distinct European Animal Families
Source: PLoS One. 2014 Dec 22;9(12):e115575. doi: 10.1371/journal.pone.0115575 (PMC4274031; doi:10.1371/journal.pone.0115575)
Supplement: S2 Table — Comparison of base composition of PCR fragments within each animal group using the reference sequences. Sequence differences are provided by annotation of the position and nucleotide within the reference sequence of species 1 in relation to position and nucleotide of species 2. Due to the change in GC content, causing different amounts of double and triple bonds, the hypothetical difference in Tm is given and compared to the observed Tm. (PDF) [file pone.0115575.s005.pdf]

Table S2

		2 → 3 bond	3 → 2 bond	Insertion	Deletion	Neutral	Theoretical T_m	Real T_m	Differences
Domestic Cattle> Yak*	12S	0	1	0	0	0	Cattle>Yak	Cattle>Yak	760C>142T
	12S universal	3	6	0	0	0	Cattle>Yak	Cattle>Yak	709G>91A; 726T>108C; 737T>119C; 739C>121T; 740C>122T; 756C>138T; 760C>142T; 817T>199C; 824C>206T
	CYTB	4	2	0	0	0	Cattle<Yak	Cattle<Yak	15002A>81G; 15005C>84T; 15011C>90T; 15014A>93G; 15038T>117C; 15059T>138C
Domestic Cattle> Zebu	12S	1	0	0	0	0	Cattle<Zebu	Cattle<Zebu	761T>332C
	12S universal	3	1	0	0	0	Cattle<Zebu	Cattle<Zebu	722C>293T; 737T>308C; 761T>332C; 816A>387G
	CYTB	0	0	0	0	0	Cattle=Zebu	Cattle=Zebu	no differences
Dog>Fox	12S (dark red primer)	2	4 +1	0	0	4 +3	Dog>Fox	Dog>Fox	370C>370T; 377C>377A; 378T>378C; 379T>379A; 381T>381A; 382A>382T; 383C>383T; 426T>426C; 436A>436T; 439C>439T; 455T>455A; 459A>459T; 460A>460T; 466C>466T
	12S universal	4	5	0	0	7	Dog>Fox	Dog</>Fox	339A>339G; 352T>352C; 370C>370T; 377C>377A; 378T>378C; 379T>379A; 381T>381A; 382A>382T; 383C>383T; 426T>426C; 436A>436T; 439C>439T; 455T>455A; 459A>459T; 460A>460T; 466C>466T
	CYTB (dark red primer)	3	4 +2	0	0	1 +1	Dog>Fox	Dog>Fox	14623T>14625A; 14632C>14634A; 14638C>14640T; 14644T>14646C; 14656T>14658C; 16659C>14661T; 14660T>14662C; 14671G>14673A; 14674C>14676T; 14680C>14682G; 14692G>14694A
Fox>Artic Fox	12S	4	1	0	0	0	Red fox< Artic fox	Red fox< Artic fox	376A>307G; 377A>308G; 380T>311C; 383T>314C; 427C>358A
	12S universal	5	4	0	0	0	Red fox< Artic fox	Fox</>Artic Fox	339G>270A; 350C>281A; 352C>283A; 376A>307G; 377A>308G; 380T>311C; 383T>314C; 427C>358A; 466T>397C
	CYTB	3	2	0	0	0	Red fox< Artic fox	Red fox< Artic fox	14649T>465C; 14659G>475A; 14676T>492C; 14682G>498A; 14691A>507G
Sheep>Goat	12S (dark red primer)	5+3	3	0	0	1	Sheep<Goat	Sheep<Goat	363G>361A; 376T>374C; 377C>375T; 379T>377C; 381C>379T; 382T>380C; 398T>396C; 426T>424C; 437A>435G; 457A>455T; 458A>456G; 459T>457C
	12S universal	8	6	0	0	1	Sheep<Goat	Sheep</>Goat	334G>332A; 351G>349A; 363G>361A; 376T>374C; 377C>375T; 379T>377C; 381C>379T; 382T>380C; 398T>396C; 426T>424C; 437A>435G; 457A>455T; 458A>456G; 459T>457C; 471C>469T
	CYTB	5	2	0	0	0	Sheep<Goat	Sheep<Goat	14653G>14647A; 14656A>14650G; 14677T>14671C; 14680C>14674T; 14701T>14695C; 14710T>14704C; 14711T>14705C
Red Deer>Roe deer	12S	8	8	0	0	1	Red deer=Roe deer	Red deer=Roe deer	361T>361C; 373C>T; 375A>C; 378C>T; 379T>A; 386A>G; 390A>C; 394T>C; 396C>T; 422G>A; 423T>C; 426C>T; 427A>G; 434T>G; 453C>A; 456C>T; 457C>A
	12S universal	9	8	0	0	1	Red deer< Roe deer	Red deer< Roe deer	349T>349C; 361T>361C; 373C>T; 375A>C; 378C>T; 379T>A; 386A>G; 390A>C; 394T>C; 396C>T; 422G>A; 423T>C; 426C>T; 427A>G; 434T>G; 453C>A; 456C>T; 457C>A
	CYTB	2	0	0	0	0	Red deer< Roe deer	Red deer< Roe deer	14686A>14675G; 14713T>14702C
Red Deer>Fallow deer	12S	7	1	0	0	1	Red deer<Fallow deer	Red deer<Fallow deer	361T>361C; 375A>375G; 423T>423C; 434T>434A; 436T>436C; 443A>443G; 449T>449C; 453C>453T; 455A>455G
	12S universal	8	2	0	0	1	Red deer<Fallow deer	Red deer<Fallow deer	345G>345A; 349T>349C; 361T>361C; 375A>375G; 423T>423C; 434T>434A; 436T>436C; 443A>443G; 449T>449C; 453C>453T; 455A>455G
	CYTB	2	3	0	0	0	Red deer> Fallow deer	Red deer> Fallow deer	14684C>14680T; 14689C>14685T; 14695T>14691C; 14716C>14712A; 14722T>14718C
Red Deer>Reindeer	12S	6	5	0	0	2	Red deer<Reindeer	Red deer<Reindeer	361T>292C; 367A>298T; 370G>301A; 376T>307C; 379T>310C; 395C>326T; 396C>327T; 421T>352C; 422G>353A; 423T>354C; 426C>357T; 427A>358G; 434T>365A
	12S universal	7	5	0	0	2	Red deer<Reindeer	Red deer<Reindeer	348A>279C; 361T>292C; 367A>298T; 370G>301A; 376T>307C; 379T>310C; 395C>326T; 396C>327T; 421T>352C; 422G>353A; 423T>354C; 426C>357T; 427A>358G; 434T>365A
	CYTB	0	3	0	0	0	Red deer>Reindeer	Red deer>Reindeer	14698C>537T; 14716C>555T; 14725C>564T
Horse>Donkey	12S	2	4	0	0	0	Horse>Donkey	Horse>Donkey	382T>383C; 383A>384C; 388C>389T; 415C>416T; 431C>432T; 443G>444A
	12S universal	4	4	1	0	0	Horse>=Donkey	Horse>=Donkey	356T>356C; d>358C; 382T>383C; 383A>384C; 388C>389T; 415C>416T; 431C>432T; 443G>444A; 473T>474C
	CYTB	0	1	0	0	1	Horse>Donkey	Horse>Donkey	14664C>14661G; 14673G>14670A
Cat > Lynx*	12S	3	3	1	2	0	Cat>=Lynx	Cat=Lynx	1240C>305T; - >82T; 1250A> -; 1251A> -; 1252T>88C; 1267T>103C; 1277T>104C; 1279C>115T; 1283G>119A
	12S universal	4	7	1	2	0	Cat>Lynx	Cat>Lynx	1215T>48C; 1240C>305T; - >82T; 1250A> -; 1251A> -; 1252T>88C; 1267T>103C; 1277T>104C; 1279C>115T; 1283G>119A; 1328C>164T; 1330C>166T; 1337C>173T; 1345C>181T
	CYTB	5	0	0	0	1	Cat<Lynx	Cat<Lynx	15547A>61G; 15562A>76G; 15565A>79C; 15576G>90C; 15592T>106C; 15601T>115C
Hare>Rabbit	12S	2	2	0	1	2	Hare>=Rabbit	Hare>Rabbit	376T>378A; 377T>379A; 378A>380C; 384G>-; 399G>400A; 413A>414G; 421C>422T
	12S universal	3	6	0	1	3	Hare>Rabbit	Hare>Rabbit	341C>443T; 349C>351T; 350T>352A; 351C>353T; 376T>378A; 377T>379A; 378A>380C; 384G>-; 399G>400A; 413A>414G; 421C>422T; 460A>461C; 461C>462T
	CYTB	0	1	0	0	1	Hare>Rabbit	Hare>Rabbit	14677C>14684T; 14689A>14696T
Pine marten/ Badger	12S	0	2	1	0	2	Pine marten> Badger	Pine marten> Badger	383C>378T; - >381T; 388A>384T; 389A>385T; 391C>387T
	12S universal	5	6	1	0	2	Pine marten>Badger	Pine marten>Badger	350T>345C; 359T>354C; 376C>371T; 383C>378T; - >381T; 388A>384T; 389A>385T; 391C>387T; 443A>439G; 464C>460T; 466T>462C; 467C>463T; 471A>467G; 473C>469T
	CYTB	3	10	0	0	0	Pine marten<Badger	not successful	14627C>14625T; 14633C>14631A; 14639C>14637T; 14647G>14645A; 14648T>14646C; 14651T>14649C; 14657G>14655A; 14669A>14667G; 14678G>14676A; 14684C>14682T; 14696A>14694C; 14699G>14697A; 14708C>14706T
Pine marten/ Otter	12S	0	3	1	0	0	Pine marten> Otter	Pine marten> Otter	381G>377A; - >385C; 391C>388T; 411C>408A
	12S universal	5	7	1	0	0	Pine marten> Otter	Pine marten> Otter	359T>355C; 376C>372T; 381G>377A; - >385C; 391C>388T; 411C>408A; 437T>434C; 445A>442G; 447C>444T; 463T>460C; 464C>461T; 467C>464A; 471A>468G
	CYTB (dark red primer)	1	6	0	0	0	Pine marten> Otter	Pine marten> Otter	14633C>14634A; 14645C>14646T; 14657G>14658A; 14678G>14679A; 14696A>14697G; 14699G>14700A; 14711C>14712T
Pine marten/ Polecat (Ferret)	12S	1	5	0	0	2	Pine marten>Polecat/Ferret	Pine marten>Polecat>Ferret (1)	383C>379T; 386C>382T; 388A>385T; 389A>385T; 391C>387T; 393C>389T; 408A>404G; 411C>407A
	12S universal	6	10	0	0	5	Pine marten>Polecat/Ferret	Pine marten>Polecat>Ferret (1)	343A>339G; 347A>343G; 351T>347C; 359T>355A; 360C>356A; 376C>372T; 383C>379T; 386C>382T; 388A>385T; 389A>385T; 391C>387T; 393C>389T; 408A>404G; 411C>407A; 416C>412T; 440A>436G; 444T>440A; 464C>460T; 466T>462C; 467C>463T; 472A>468T
	CYTB (dark red primer)	2 +1	11 +1	0	0	1	Pine marten>Polecat/Ferret	Pine marten>Polecat/Ferret	14720T>14718C; 14726G>14724A; 14624A>14622T; 14627C>14625T; 14633C>14631A; 14636C>14634T; 14645C>14643T; 14647G>14645A; 14657G>14655A; 14669A>14667C; 14672G>14670A; 14678G>14676A; 14684C>14682T; 14690C>14688T; 14699G>14697A; 14711C>14709T
Pine marten/ Stoat	12S	3	3	0	0	0	Pine marten=Stoat	Pine marten>Stoat (1)	383C>309T; 388A>314C; 389A>315C; 390A>316G; 391C>317T; 411C>337A
	12S universal	8	7	0	0	1	Pine marten<Stoat	Pine marten<Stoat	347A>273G; 351T>277C; 359T>285A; 360C>286A; 376C>302T; 383C>309T; 388A>314C; 389A>315C; 390A>316G; 391C>317T; 411C>337A; 444T>370G; 463T>389C; 464C>390T; 466T>392C; 467C>393T
	CYTB (dark red primer)	3	9	0	0	0	Pine marten>Stoat	Pine marten>Stoat	14624A>453C; 14633C>462A; 14645C>474T; 14647G>476A; 14648T>477C; 14657G>486A; 14669A>498C; 14672G>501A; 14678G>501A; 14678G>507A; 14694C>523T; 14699G>528A
Pine marten/ Beech marten	12S		1	0	0	0	Pine marten>Beech marten	Pine marten>Beech marten	383C>381T
	12S universal	2	3	0	0	0	Pine marten>Beech marten	Pine marten>Beech marten	347A>345G; 383C>381T; 437T>435C; 464C>462T; 467C>465T
	CYTB	3	3	0	0	0	Pine marten=Beech marten	not successful	14636C>14635T; 14648T>14647C; 14651T>14650C; 14657G>14656A; 14669A>14668G; 14699G>14698A
Chicken>Turkey	12S	3	6	0	0	0	Chicken>Turkey	Chicken>Turkey	1608T>379C; 1614G>385A; 1615C>386T; 1620G>391A; 1621C>392T; 1628T>399C; 1632A>403G; 1635C>406T; 1639C>410T
	12S universal	8	13	2	1	4	Chicken>Turkey	Chicken<Turkey	1582T>353C; 1584C>355T; 1601C>372T; 1608T>379C; 1614G>385A; 1615C>386T; 1620G>391A; 1621C>392T; 1628T>399C; 1632A>403G; 1635C>406T; 1639C>410T; 1655T>426C; 1661C>433T; 1669A>440G; 1674A>445G; 1677C>448T; - >451A; - >452A; 1681T>454A; 1682C>455T; 1683C>456G; 1690G>463A; 1692C<d; 1694C>466T; 1701T>473C; 1707T>479A; 1710C>482G
	CYTB	3	3	0	0	1	Chicken=Turkey	Chicken>=Turkey	15354T>14132C; 15366A>14144T; 15369C>14147A; 15381G>14159A; 15384A>14162G; 15399T>14177C; 15405C>14183A

* no NCBI ref, comparison to consensus sequence of samples

(1) Differences due to mutations within the samples not seen within the reference sequence of NCBI
